# Single-cell transcriptomics reveals a correlation between inflammatory C7 fibroblasts in cervical tumor-adjacent normal tissues and cervical cancer progression

**DOI:** 10.3389/fcell.2026.1829843

**Published:** 2026-08-10

**Authors:** Yi Liu, Dingling Yin, Lu Wu, Huan Wang, Yijun Song, Jie Huang, Jiaxiong Zhou, Haoxian Li, Peili Liang, Jinghui Feng, Hong He

**Affiliations:** 1 Guangdong Provincial Key Laboratory of Major Obstetric Diseases, Guangdong-Hong Kong-Macao Greater Bay Area Higher Education Joint Laboratory of Maternal-Fetal Medicine, Department of Obstetrics and Gynecology, Guangdong Provincial Clinical Research Center for Obstetrics and Gynecology, The Third Affiliated Hospital, Guangzhou Medical University, Guangzhou, China; 2 School of Biomedical Engineering, Guangzhou Medical University, Guangzhou, China

**Keywords:** C7 fibroblasts, cervical cancer, inflammatory cancer-associated fibroblasts, tumor microenvironment, tumor-adjacent normal tissue

## Abstract

**Introduction:**

Cervical cancer is a common gynecological malignancy, having high incidence and mortality rates, especially in developing regions. The tumor microenvironment (TME) plays a crucial role in disease progression, and cancer‐associated fibroblasts (CAFs) are key components of the TME. However, the roles of CAFs in tumor‐adjacent normal tissue remain unclear.

**Methods:**

Herein, we used single‐cell RNA sequencing to generate high‐resolution cellular atlases of cervical cancer and tumor‐adjacent normal tissue, systematically profiling fibroblast–cancer cell interactions and comparing fibroblasts from distinct origins. Pseudotime analysis and CytoTRACE scoring were employed to investigate the developmental trajectory of fibroblasts. Immunohistochemistry validated the inflammatory CAF (iCAF) subpopulation of C7 fibroblasts and assessed its correlations with clinicopathological features.

**Results:**

We found that C7 fibroblasts secrete CXCL12 to activate the ACKR3 (CXCR7) receptor in cancer cells, thereby promoting tumor cell proliferation, invasion, and metastasis. C7 fibroblasts also highly expressed multiple oncogenic genes, including IL6, CXCL3, CXCL2, CCL2, GPC3, PLAU, TNFAIP6, PTX3, and EIF4A3, and genes associated with poor prognosis, including CXCL3, TNFAIP6, PTX3, IGSF10, and LDLR. High C7 fibroblast abundance was associated with advanced International Federation of Gynecology and Obstetrics stage, lymph node metastasis, and postmenopausal status.

**Discussion:**

Our findings suggest that C7 fibroblasts being predominantly distributed in tumor‐adjacent normal tissues is significantly associated with cervical cancer progression, and that C7 fibroblasts in these tissues may serve as potential biomarkers and therapeutic targets for tumor microenvironment modulation.

## 1 Introduction

Cervical cancer is a common gynecological tumor worldwide, posing a serious threat to the life and health of women worldwide (Bhatla et al., 2021). Despite the significant progress made in disease prevention through the popularization of cervical cancer screening and the launch of human papillomavirus (HPV) vaccines, the incidence of cervical cancer remains high (Li et al., 2024; Cao et al., 2023). According to a report by the World Health Organization (WHO), approximately 604,000 new cases of cervical cancer and 342,000 deaths occurred worldwide in 2020 (Sung et al., 2021). With the deepening of research, more evidence indicates that the progression of cervical cancer is not solely determined by the intrinsic characteristics of cancer cells but is profoundly influenced by the tumor microenvironment (TME) (Trujillo-Cirilo et al., 2023; Li et al., 2023; Zhou et al., 2022).

The TME is a complex environment of tumor cells, encompassing blood vessels, immune cells, fibroblasts, and bone marrow-derived inflammatory cells, as well as the bioactive molecules they secrete (Xia et al., 2023). These components interact and play an important role in tumorigenesis and development (Cao et al., 2021; Ho et al., 2020). Cancer-associated fibroblasts (CAFs) are core components in the TME of cervical cancer and play a key role in tumor progression, immune escape, and poor prognosis (Li et al., 2022; Lin et al., 2025; Yang et al., 2020; Wei et al., 2023). However, existing studies mostly focus on CAFs within tumor lesions, while paying insufficient attention to those in tumor-adjacent normal tissue.

Therefore, in this study, we aimed to systematically characterize the cellular landscape of cervical cancer and its tumor-adjacent normal tissue, with a particular focus on fibroblast populations within the tumor microenvironment. By integrating single-cell transcriptomics analysis with the immunohistochemical validation of clinical samples, we sought to investigate the interactions between fibroblasts and cervical cancer cells in both tumor and adjacent normal tissues, as well as to explore the relationship between fibroblasts in tumor-adjacent normal tissue and patient clinical characteristics. This study was designed to elucidate the heterogeneity of fibroblasts within the tumor microenvironment and to investigate the potential contribution of fibroblasts in tumor-adjacent normal tissue to cervical cancer progression, thereby providing new insights into the complexity of the tumor microenvironment and informing the exploration of potential therapeutic targets for cervical cancer.

## 2 Methods

### 2.1 Ethical approval

This study was approved by the Ethics Committee of the Third Affiliated Hospital of Guangzhou Medical University (Approval Number: MedEthics Committee Review [2023] No. 57). All the tissues were obtained from the biobank of the Third Affiliated Hospital of Guangzhou Medical University. All patients who provided samples for single-cell sequencing and immunohistochemistry experiments provided written informed consent. Moreover, this research was conducted strictly in accordance with the ethical principles outlined in the Helsinki Declaration (1975, and subsequent revisions).

### 2.2 Clinical specimen collection

This study collected tissue samples from four cervical cancer patients, including four cancer tissue samples and three tumor-adjacent normal tissue samples. After collection, all the samples were immediately immersed in tissue preservation solution to maintain the tissue’s activity. The histological diagnoses of all patients were confirmed as cervical squamous cell carcinoma through standard histopathological assessment for single-cell sequencing. Additionally, we obtained cancer tissue and matched tumor-adjacent normal tissue samples from 12 patients with cervical squamous cell carcinoma from the biobank of the Third Affiliated Hospital of Guangzhou Medical University. These samples were used for immunohistochemical staining to evaluate the presence and relative abundance of C7 fibroblasts in both cancer tissues and tumor-adjacent normal tissues. Furthermore, we collected tumor-adjacent normal tissue samples from 81 patients with cervical squamous cell carcinoma from the same biobank to assess the association between the abundance of C7 fibroblasts in tumor-adjacent normal tissues and patients' clinicopathological characteristics.

In this study, “tumor-adjacent normal tissue” refers to a small piece of normal tissue excised during surgery that is immediately adjacent to the tumor edge and directly contiguous with the main tumor body. Preoperative imaging, colposcopy, biopsy, and clinical evaluation were used to define the tumor extent. Intraoperatively, after resecting tumor tissue within the lesion scope, surgeons simultaneously excised an adjacent tissue specimen contiguous with the tumor edge as a margin specimen for routine histopathological assessment to confirm a negative resection margin and complete tumor removal. This margin tissue was verified by pathology to show no evidence of tumor cell infiltration or invasion and exhibited normal cervical histology; therefore, it was defined as “tumor-adjacent normal tissue.” Importantly, this tissue is anatomically adjacent to the tumor and represents the peritumoral microenvironment rather than distant normal tissue.

### 2.3 Sample processing and single-cell RNA sequencing

The collected tissue specimens were placed in sample transport containers maintained on ice and promptly delivered to Aoke Biological Co., Ltd. For single-cell RNA sequencing. All sequencing procedures were conducted in strict accordance with the manufacturer’s instructions and the standard operating protocols of the 10× Genomics platform.

### 2.4 scRNA-seq data preprocessing

The scRNA-seq data were analyzed using the R package “Seurat” (version 5.2.0) in R (version 4.4.2). Cell filtering was conducted using the following criteria: number of detected genes per cell (nFeature_RNA) between 200 and 7,000; proportion of mitochondrial genes (mt_percent) less than 20%; proportion of erythrocyte genes (HB_percent) less than 5%; total UMI count per cell (nCount_RNA) greater than 1,000; and exclusion of the top 3% of cells with the highest UMI counts. Data normalization was performed using the “NormalizeData ()” function, followed by identification of highly variable genes using the “FindVariableFeatures ()” function. Data scaling was conducted with the “ScaleData ()” function, during which mt_percent was regressed out to reduce the influence of mitochondrial gene expression. Principal component analysis (PCA) was then performed using the “RunPCA()” function.

Batch effect correction was performed using the Harmony algorithm implemented in Seurat v5 through the “IntegrateLayers ()” function with HarmonyIntegration. The PCA reduction was used as the input, and the Harmony-corrected embeddings were saved as a new reduction named “harmony.” The main parameters were set as follows: orig. reduction = “pca,” new. reduction = “harmony,” and verbose = FALSE. Other Harmony-related parameters were kept at their default settings in Seurat version 5.2.0, including theta = NULL, lambda = NULL, sigma = 0.1, nclust = NULL, tau = 0, block. size = 0.05, max. iter.harmony = 10, and max. iter.cluster = 20.

To validate the effectiveness of batch effect correction, PCA and UMAP distributions before and after Harmony integration were compared, with cells colored by sample source and tissue type. Before correction, cells showed partial separation according to sample or tissue origin, whereas after Harmony integration, cells from different samples were better mixed while major biological cell populations remained distinguishable. These results indicated that Harmony integration effectively reduced technical variation while preserving biological heterogeneity.

The cell cycle scores for the G2/M and S phases were calculated using “CellCycleScoring ()” function based on known cell cycle signature genes. The cell cycle labels were annotated accordingly. The sample group metadata were integrated into the Seurat object using “left_join ()” function. The processed Seurat object was saved for subsequent analyses.

### 2.5 Cell annotation

To identify distinct cellular populations, single-cell RNA sequencing data were analyzed using the “Seurat” R package (version 5.2.0). A shared nearest neighbor (SNN) graph was constructed based on the Harmony-corrected expression matrix using the “FindNeighbors ()” function with default parameters, followed by unsupervised clustering with the “FindClusters ()” function to partition cells into distinct clusters.

To identify cluster-specific marker genes, the “FindAllMarkers ()” function was applied with the following parameters: test. use = “wilcox,” using the Wilcoxon rank-sum test, a non-parametric method commonly used for identifying differentially expressed genes; slot = “data,” using normalized data instead of raw counts to reduce technical bias due to sequencing depth; min. pct = 0.25, requiring genes to express in at least 25% of cells to ensure representativeness of the cluster; and logfc. threshold = 0.25, selecting genes with at least a 0.25 log2 fold change between clusters to ensure biological relevance.

The initially identified marker genes were further filtered using the following criteria: pct.1 > 0.1, indicating that the gene must be expressed in at least 10% of cells within the target cluster to ensure representativeness; p_val_adj <0.05, applying an adjusted p-value to control for false positives and ensuring statistical significance; and avg_log2FC > 2, ensuring a strong fold change in gene expression between the target cluster and others. These criteria ensured that the selected genes were significantly expressed in the target cluster with a sufficient proportion of expression.

For each cluster, the top 10 significantly differentially expressed genes were selected as representative marker genes for cell type annotation. Cell type annotation was performed using the CellMarker 2.0 database (https://bio-bigdata.hrbmu.edu.cn/CellMarker/), a comprehensive resource of human and mouse cell markers derived from single-cell RNA sequencing data.

To validate the annotation accuracy, the expression patterns of known cell type markers were further examined within the corresponding clusters. The “FeaturePlot ()” function was used to visualize the spatial expression of these canonical marker genes, confirming consistency with the assigned cell type and thereby enhancing the accuracy of annotation.

### 2.6 Cell communication analysis

Cell-cell communication analysis was conducted using the “CellChat” R package (version 1.6.1). Initial data integration was performed using “FindIntegrationAnchors ()” and “IntegrateData (),” followed by dimensionality reduction and clustering. A CellChat object was constructed using the “createCellChat ()” function, with the CellChatDB.human database employed to infer ligand-receptor interactions. The intercellular communication probabilities were computed using “computeCommunProb (),” allowing the identification of high-frequency communication pairs. Pathway-specific communication was assessed using “computeCommunProbPathway ().” Finally, “netAnalysis_signalingRole_network ()” was used to investigate the signaling roles of individual cell types within the cell communication network.

### 2.7 Identification of differentially expressed genes (DEGs)

The “FindAllMarkers ()” was used to detect DEGs in each cell cluster, and the Wilcoxon test was used to identify the significantly expressed genes in the cells. The screening criteria included an adjusted p-value (p_val_adj <0.05) and an average log2 fold change (|avg_log2FC| > 0).

### 2.8 The cancer genome atlas (TCGA) prognostic analysis

Gene expression data for cervical cancer were collected from TCGA repository (http://cancergenome.nih.gov/cancergenomics/tissuesamples), which can be downloaded from the UCSC website (https://xenabrowser.net/). The TCGA data from UCSC Xena already underwent standardized preprocessing; therefore, no additional data filtering or normalization steps were needed in our study. Prognostic analysis was performed using R (version 4.4.2), primarily utilizing the “survival” (version 3.8.3) and “survminer” (version 0.5.0) R packages. After constructing the Surv object, patients were divided into high- and low-expression groups based on the median gene expression value. Survival curves were generated using the Kaplan-Meier method, and the log-rank test was applied to compare survival differences between the two groups. The p-value and median survival time were calculated and reported. The Cox proportional hazards regression model was employed to estimate the hazard ratios (HRs) and 95% confidence intervals (CIs) for both high- and low-expression groups, with HR values and p-values annotated on the survival plots.

Notably, for the prognostic analysis of cervical cancer using the TCGA dataset, only gene expression data from cervical squamous cell carcinoma were included in this study. This ensured consistency with our single-cell sequencing data, which were also derived exclusively from cervical squamous cell carcinoma, as the C7 fibroblast-related gene signature identified in the current work was obtained from this pathological subtype.

### 2.9 Pseudotime analysis

Pseudotime analysis was performed using the “Monocle 2” R package (version 2.34.0). First, data preprocessing involved subsetting fibroblast subtypes of interest and creating a Monocle object from the gene expression matrix. The “newCellDataSet ()” function was used to construct a cell dataset, followed by the “estimateSizeFactors ()” and “estimateDispersions ()” functions for normalization and dispersion estimation, respectively. Next, differential gene testing was conducted using the “differentialGeneTest ()” to identify genes associated with the differentiation process, with the top 1,000 genes selected for ordering. Trajectory analysis was performed using dimensionality reduction through the “reduceDimension ()” function with the DDRTree method, followed by cell ordering using “orderCells ().” Finally, the developmental trajectory was visualized with the “plot_cell_trajectory (),” showing cell states, cell types, and pseudotimes.

### 2.10 CytoTRACE analysis

CytoTRACE scores were calculated using the “CytoTRACE” R package (version 0.3.3) to estimate the differentiation potential of individual cells. First, single-cell RNA-seq data was preprocessed by filtering out genes that expressed in fewer than five cells. The RNA count matrix was extracted from the Seurat object using the “GetAssayData ()” function. The CytoTRACE function was then applied to the filtered expression matrix, with “ncores = 1” to specify the use of a single CPU core for the computation. Cell-type annotations were extracted from the metadata and used for phenotype assignment. UMAP embeddings were also extracted for visualization. The results were visualized using the “plotCytoTRACE ()” function.

### 2.11 Immunohistochemistry

#### 2.11.1 Immunohistochemical objective

To investigate differences in the abundance of C7 fibroblasts between cervical squamous cell carcinoma tissue (cancer tissue) and the corresponding tumor-adjacent normal tissue (matched tumor-adjacent normal tissue), we performed immunohistochemical staining on paired samples from 12 patients. We selected paired samples from 12 cervical cancer patients for analysis. Specifically, because clinical tissue samples are difficult to obtain and highly valuable. While ensuring that the sample size was sufficient for statistical analysis, we conducted preliminary validation in the small cohort of 12 paired samples. This strategy ensured the accuracy of our research findings while minimizing the use of precious clinical samples. Fibroblast activation protein (FAP), a fibroblast marker, and C7, a marker of C7 fibroblasts, were selected as detection indicators. To minimize the influence of overall differences in the number of fibroblasts on the results, we first identified regions with comparable FAP staining intensity and distribution in both types of tissues. Comparable FAP staining intensity indicated that the content of fibroblasts in the corresponding regions was comparable. Further analysis of C7 staining intensity within these regions was conducted to assess the relative content difference of C7 fibroblasts in cancer tissue and adjacent normal tissue.

To further investigate the relationship between the abundance of C7 fibroblasts in tumor-adjacent normal tissues and the clinical manifestations of patients with cervical squamous cell carcinoma, we expanded the sample size based on our preliminary findings and selected tumor-adjacent normal tissue samples from 81 cervical cancer patients as a large cohort for immunohistochemical analysis. The detection indicator was C7 (C7 fibroblast marker). By analyzing the staining intensity of C7, we evaluated the abundance of C7 fibroblasts in the tumor-adjacent normal tissues and its relationship with the clinical manifestations of the patients.

The specific clinical subgroups of cervical cancer patients were defined based on distinct clinical and pathological features, which included tumor differentiation grade, International Federation of Gynecology and Obstetrics (FIGO) stage, lymph node metastasis (LN) status, lymphovascular invasion (LVI) status, perineural invasion (PNI) status, abnormal uterine bleeding (AUB) status, menopausal status, and human papillomavirus (HPV) status. The significance of these features is as follows: tumor differentiation grade and FIGO stage were used to explore the correlation between C7 fibroblasts and metastasis; AUB status was used to examine the association between C7 fibroblasts and the risk of AUB in cervical cancer; and menopausal status was used to determine whether C7 fibroblasts were involved in the development of both HPV-related and non-HPV-related cervical cancer.

#### 2.11.2 Immunohistochemical staining procedure

The tissue samples were embedded in paraffin and cut into 4-μm-thick tissue sections. The samples were dewaxed with xylene and then hydrated with ethanol and distilled water. The slides were heated for antigen retrieval as follows: sodium citrate buffer (pH 6) for C7 and Tris-EDTA buffer (pH 9) for fibroblast activation protein (FAP). Endogenous peroxidase activity was inhibited by incubation with 3% hydrogen peroxide for 10 min. Subsequently, the sections were incubated with the following primary antibodies: anti-FAP (1:200; 11779-1-AP, Proteintech) and anti-C7 (1:100; #DF9410, Affinity). Tissue sections were washed thrice with phosphate-buffered saline (PBS), incubated with the appropriate horseradish peroxidase-conjugated secondary antibodies for 1 h at room temperature, and detected using the 3,3′-diaminobenzidine (DAB) Substrate Kit (SK-4100, Vector). After dehydration through a graded ethanol series, the sections were cleared in xylene for transparency, before mounting with a resinous medium.

#### 2.11.3 Immunohistochemical imaging

For the paired samples from 12 patients with cervical squamous cell carcinoma, the tissue sections were observed and images were captured under a 40× microscope to identify regions where FAP expression was relatively consistent. Notably, regions with comparable FAP staining intensity were selected for C7 expression analysis. Because FAP serves as a marker for fibroblasts, while C7 is a specific marker for C7 fibroblasts, selecting such regions ensures equivalent total fibroblast content in the analyzed areas between cancer tissue and tumor-adjacent normal tissue. On this basis, comparison of C7 expression levels allows for the accurate assessment of the relative abundance of C7 fibroblasts between the two tissue groups.

For the tumor-adjacent normal tissue samples from 81 patients with cervical squamous cell carcinoma, the tissue sections were observed and imaged under a 15× microscope. The larger field of view enabled better evaluation of the distribution of C7 fibroblasts in the tumor-adjacent normal tissues, thereby reducing errors caused by the deviation in sample area selection and improving the accuracy and reliability of the analysis.

To minimize selection bias, three non-overlapping fields were randomly selected for each tissue section under consistent microscopic parameters. Image acquisition and quantitative analysis were performed in a blinded manner by two researchers who were unaware of the sample grouping information, ensuring their objectivity and reducing potential subjective bias.

#### 2.11.4 Quantitative immunohistochemical analysis

In this study, quantitative immunohistochemical analysis was performed using ImageJ software. The images were analyzed using a uniform threshold for batch processing to minimize errors. The normalization analysis procedure was as follows: 1. A new folder was created to store all images to be analyzed; 2. ImageJ was opened, after which the sequence Plugins → Macros → Record was used to record the operation code; 3. The first image was dragged from the folder into the toolbar, followed by clicking Image → Colour → Colour Deconvolution → H DAB → OK; 4. From the three deconvolved images, the one with the red-brown color was kept; 5. The sequence Analyze → Calibrate → Uncalibrated OD was used to check the Global Calibration and show the plot, and then OK was clicked; 6. The threshold was adjusted as follows: Image → Adjust → Threshold. The original image and the red-brown image were compared together, and the red-brown image was clicked on and the sliding module adjusted to ensure that the threshold range covered the positive areas; 7. The Analyze → Set Measurements sequence was used to check the required parameters; 8. The Analyze → Measure sequence was used to obtain the analysis results of the image, after which the operation code was saved; 9. ImageJ was reopened, followed by clicking through the Process → Batch → Macro sequence to open the operation code. The folder with the images was selected in Input, and then Test was clicked on to obtain the quantitative analysis results for all images.

### 2.12 Statistical analysis

In this study, statistical analysis and data visualization were performed using GraphPad Prism software (version 10.2.3). The normality of the data distribution was first assessed using the Shapiro–Wilk test, and homogeneity of variance among groups was evaluated using Levene’s test. After confirming that the data met these assumptions, a two-tailed independent samples t-test was used for comparisons between two groups. One-way ANOVA was applied to assess differences among multiple groups, followed by Tukey’s multiple comparisons test for pairwise *post hoc* analyses when statistical significance was observed. Unless otherwise stated, *p* < 0.05 was considered statistically significant.

## 3 Results

### 3.1 Single-cell transcriptomics of cervical cancer and tumor-adjacent normal tissue

**FIGURE 1:**
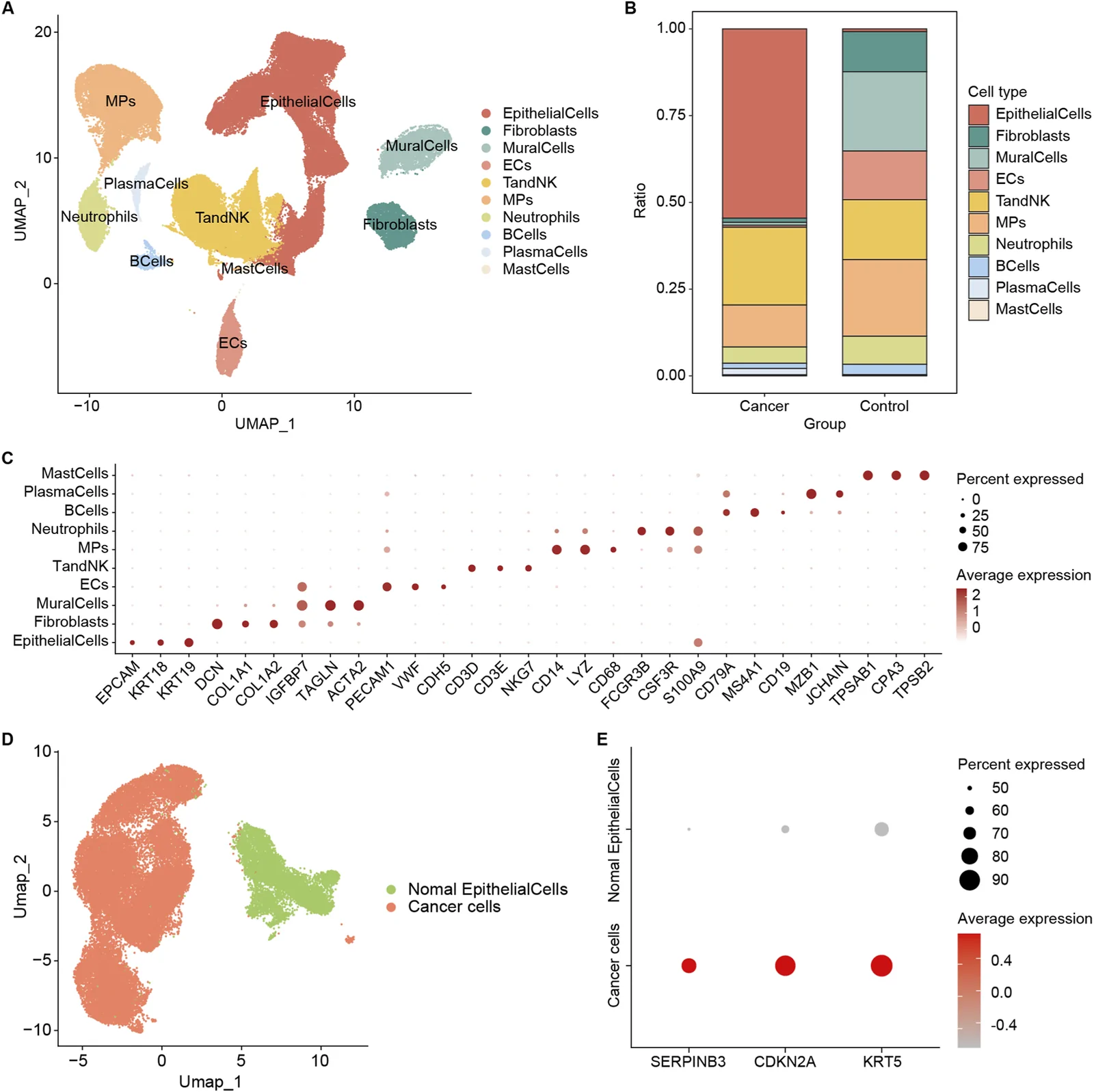
Single-cell transcriptomic landscape of cervical cancer and tumor-adjacent normal tissue. **(A)** Uniform manifold approximation and projection (UMAP) visualization delineating distinct cell populations, with each cell type indicated by a specific color; **(B)** Cell proportion chart illustrating the percentage distribution of various cell types in cervical cancer tissues (Cancer) and adjacent normal tissues (Control); **(C)** Bubble plot visualizing the expression levels of specific markers in each identified cell type; **(D)** UMAP plot delineating cancer cells and normal epithelial cells within cancer tissues; **(E)** Bubble plot showing the expression of cervical cancer marker genes SERPINB3, KRT5, and CDKN2A in cancer cells and normal epithelial cells within cancer tissues.

To determine the cellular characteristics of cervical cancer tissues and their adjacent normal tissues, we collected seven clinical samples for single-cell RNA sequencing analysis, including four cases of cervical cancer tissues (cancer) and three cases of tumor-adjacent normal tissues (control). After strict quality control screening, a total of 84,504 high-quality cells were obtained for subsequent research. The cancer tissue samples contained 63,782 cells, and the adjacent normal tissue samples contained 20,722 cells. Based on the highly expressed gene characteristics and classical cell markers of each cell cluster (Cao et al., 2023; Li et al., 2022), we classified these cells into ten major types (Figures 1A,C): Epithelial cells (n = 34,958), fibroblasts (n = 3,161), mural cells (n = 5,264), endothelial cells (ECs; n = 3,277), T and NK cells (n = 17,866), mononuclear phagocytes (MPs; n = 12,296), neutrophils (n = 4,669), B cells (n = 1,568), plasma cells (n = 1,206), and mast cells (n = 239). The cell ratio chart showed that the cervical cancer tissue (cancer) was mainly composed of epithelial cells and T/NK cells, while the tumor-adjacent normal tissue (control) was mainly composed of fibroblasts, mural cells, and MPs (Figure 1B). Subsequently, based on the characteristic marker genes of cervical cancer cells, including *SERPINB3*, *KRT5*, and *CDKN2A* (Ou et al., 2022; Sun et al., 2017), we further distinguished the epithelial cells within the cancer tissue into cancer and normal epithelial cells (Figures 1D–F).

### 3.2 Fibroblasts in tumor-adjacent normal tissues promote cervical cancer progression via the CXCL12–ACKR3 signaling axis

**FIGURE 2:**
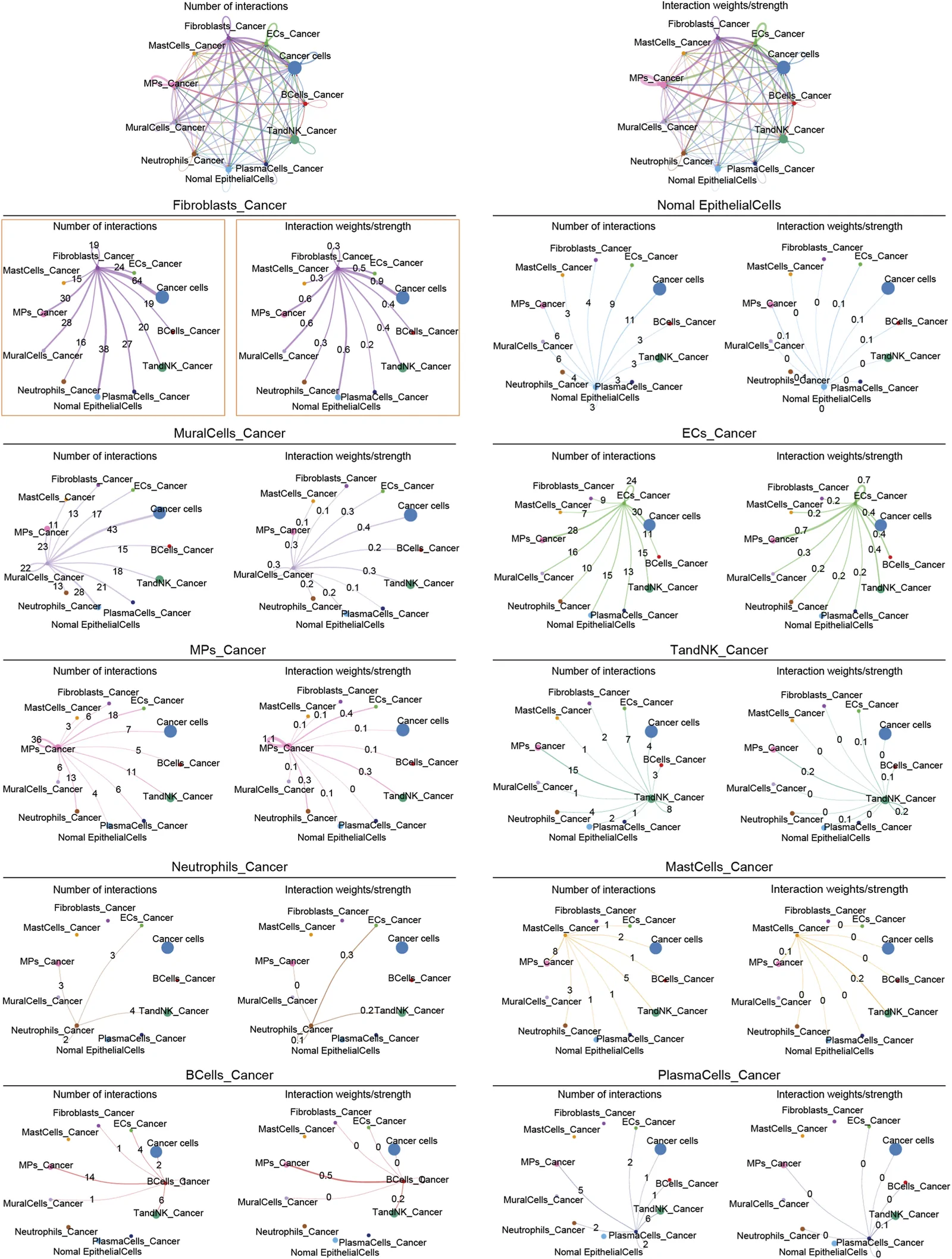
Cell–cell interaction plot illustrating the number and strength of interactions within cancer tissues between various cell types sending signals and cancer cells receiving signals. These results focus only on interactions in which cancer cells functioned as signal receivers. Specifically, Fibroblasts_Cancer (fibroblasts within cancer tissues) → Cancer cells: number = 64, strength = 0.9; Normal EpithelialCells_Cancer (normal epithelial cells within cancer tissues) → Cancer cells: number = 11, strength = 0.1; MuralCells_Cancer (mural cells within cancer tissues) → Cancer cells: number = 43, strength = 0.4; ECs_Cancer (endothelial cells within cancer tissues) → Cancer cells: number = 30, strength = 0.4; MPs_Cancer (mononuclear phagocytes within cancer tissues) → Cancer cells: number = 7, strength = 0.1; TandNK_Cancer (T and NK cells within cancer tissues) → Cancer cells: number = 4, strength = 0; Neutrophils_Cancer (neutrophils within cancer tissues) → Cancer cells: no detected interaction; MastCells_Cancer (mast cells within cancer tissues) → Cancer cells: number = 2, strength = 0; BCells_Cancer (B cells within cancer tissues) → Cancer cells: number = 2, strength = 0; and PlasmaCells_Cancer (plasma cells within cancer tissues) → Cancer cells: number = 1, strength = 0.

**FIGURE 3:**
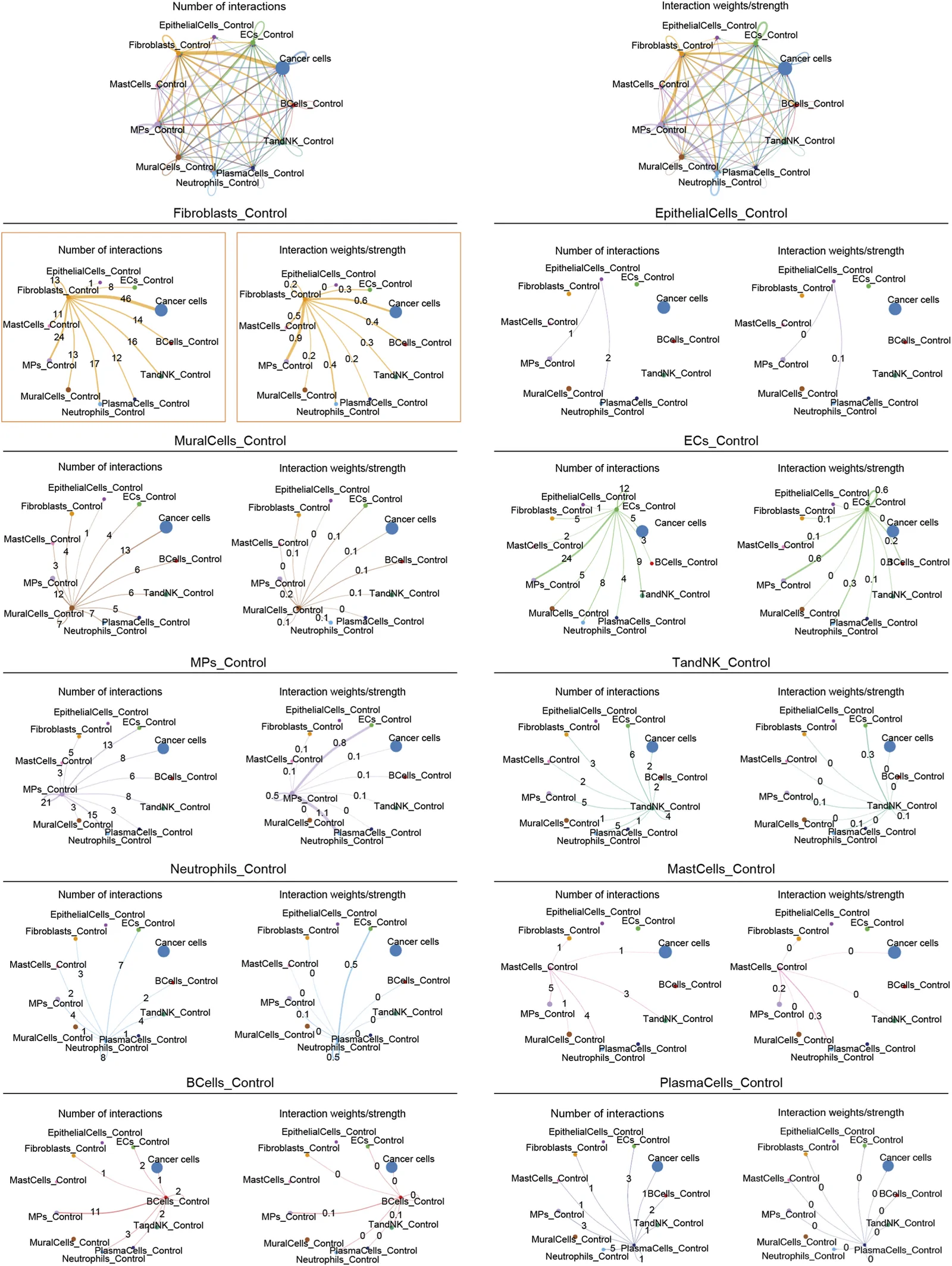
Cell–cell interaction plot illustrating the number and strength of interactions within tumor-adjacent normal tissues between various cell types sending signals and cancer cells receiving signals. These results focus only on interactions in which cancer cells functioned as signal receivers. Specifically, Fibroblasts_Control (fibroblasts within adjacent normal tissues) → Cancer cells: number = 46, strength = 0.6; EpithelialCells_Control (epithelial cells within adjacent normal tissues) → Cancer cells: no detected interaction; MuralCells_Control (mural cells within adjacent normal tissues) → Cancer cells: number = 13, strength = 0.1; ECs_Control (endothelial cells within adjacent normal tissues) → Cancer cells: number = 5, strength = 0; MPs_Control (mononuclear phagocytes within adjacent normal tissues) → Cancer cells: number = 8, strength = 0.1; TandNK_Control (T and NK cells within adjacent normal tissues) → Cancer cells: number = 2, strength = 0; Neutrophils_Control (neutrophils within adjacent normal tissues) → Cancer cells: no detected interaction; MastCells_Control (mast cells within adjacent normal tissues) → Cancer cells: number = 1, strength = 0; BCells_Control (B cells within adjacent normal tissues) → Cancer cells: number = 1, strength = 0; PlasmaCells_Control (plasma cells within adjacent normal tissues) → Cancer cells: number = 1, strength = 0.

**FIGURE 4:**
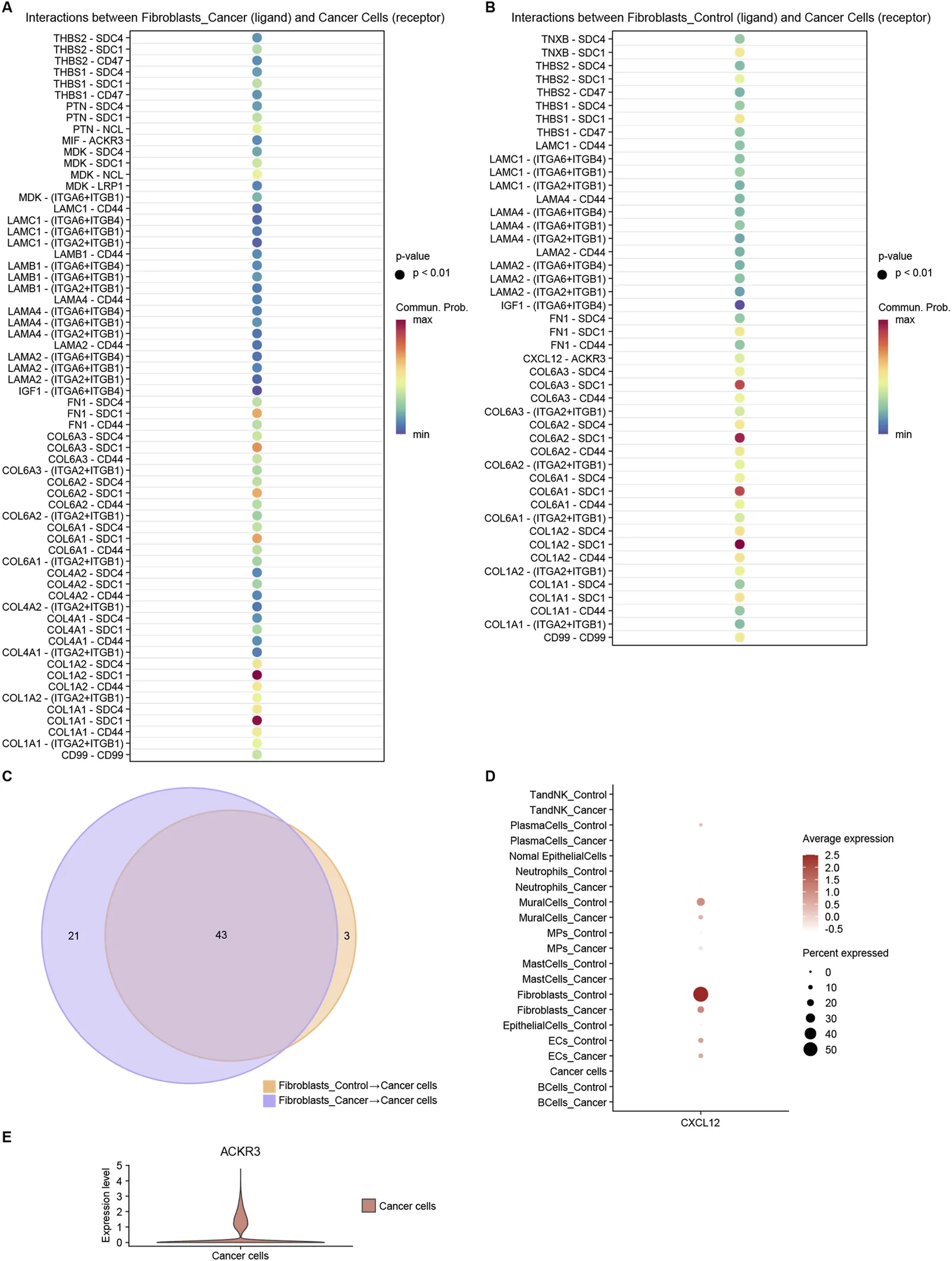
Cell–cell interaction analysis between fibroblasts and cancer cells, and expression levels of CXCL12 across different cell types. **(A)** A total of 64 ligand–receptor interaction gene pairs between Fibroblasts_Cancer (fibroblasts from cancer tissues) and cancer cells; **(B)** 46 ligand–receptor interaction gene pairs between Fibroblasts_Control (fibroblasts from adjacent normal tissues) and cancer cells; **(C)** Venn diagram showing shared and unique ligand–receptor interaction gene pairs between fibroblasts of different histological origins and cancer cells; **(D)** Bubble plot showing the abundance and expression levels of the CXCL12 gene across different cell types; **(E)** Violin plot showing ACKR3 expression levels in cervical cancer cells.

To investigate the potential roles of different cell types within the TME in the initiation and progression of cervical cancer, we systematically analyzed the cell–cell interactions between various cell types and cancer cells in cancer and adjacent normal tissues. Fibroblasts exhibited the highest number and strongest intensity of interactions with cancer cells in both tumor and adjacent normal tissues (Figures 2, 3). A total of 64 ligand-receptor interaction gene pairs were identified between fibroblasts from cancer tissues and cells (Figures 2, 4A; Supplementary Table S1). A total of 46 interaction gene pairs were identified between fibroblasts from adjacent normal tissues and cancer cells (Figures 3, 4B; Supplementary Table S2). Among these, 43 ligand-receptor gene pairs were shared, 21 were specific to fibroblasts from cancer tissues and cells, and three interaction gene pairs were specific to fibroblasts from adjacent normal tissues and cancer cells (Figure 4C; Supplementary Table S3). Our findings suggest that fibroblasts can maintain extensive interactions with cervical cancer cells across different tissue contexts.

Among the 21 unique ligand–receptor interaction gene pairs between fibroblasts from cancer tissues and cancer cells identified in this study, previous research has demonstrated that the MDK–SDC1 axis can activate the PI3K/AKT and p38 MAPK signaling pathways, thereby significantly promoting tumor cell proliferation, migration, and invasion, as well as inducing lymphangiogenesis and synergistically driving lymph node metastasis (Lin et al., 2025; Fei et al., 2024). The MIF–ACKR3 (CXCR7) axis has been shown to enhance vascular endothelial growth factor (VEGF) secretion and is strongly associated with an increased lymph node metastasis rate, higher recurrence rate, and poorer prognosis (Cui et al., 2025; Cheng et al., 2011; Krockenberger et al., 2010; Wang et al., 2017; Schrevel et al., 2012; Zhao et al., 2019). These 21 gene pairs represent interactions specific to tumor-assisted fibroblasts, distinguishing them from fibroblasts in tumor-adjacent normal tissues. Notably, MDK–SDC1 and MIF–ACKR3 (CXCR7) are included in this set, indicating that MDK and MIF are specifically secreted by fibroblasts in tumor tissues and act on their corresponding receptors on cancer cells, while such interactions are absent in adjacent normal tissues. While our study did not initially hypothesize the functional roles of MDK–SDC1 or MIF–ACKR3 in cervical cancer progression, highlighting these previously reported interactions supports the biological relevance of our bioinformatics findings and reinforces the potential contribution of tumor-assisted fibroblasts to cervical cancer progression.

Notably, among the three unique ligand–receptor interaction gene pairs identified exclusively between fibroblasts from adjacent normal tissues and cancer cells, including CXCL12–ACKR3 (CXCR7), previous studies have shown that the CXCL12–ACKR3 (CXCR7) axis can enhance the adhesion of cervical cancer cells and upregulate the expression of matrix metalloproteinases MMP2 and MMP9 via an ACKR3-dependent mechanism, thereby promoting cell proliferation, invasion, and metastasis (Xu et al., 2021). While our bioinformatics analysis did not directly identify a tumor-promoting role for this gene pair, its inclusion highlights a fibroblast–cancer cell interaction specifically present in adjacent normal tissues. CXCL12 was highly expressed exclusively in fibroblasts from adjacent normal tissues (Figure 4D), while ACKR3 was expressed in cervical cancer cells (Figure 4E). These findings indicate that fibroblasts within adjacent normal tissues may play a pivotal role in shaping the malignant microenvironment and promoting cervical cancer progression.

### 3.3 C7 fibroblasts are predominantly located in tumor-adjacent normal tissues, show high CXCL12 expression, and correspond to iCAF subtype

**FIGURE 5:**
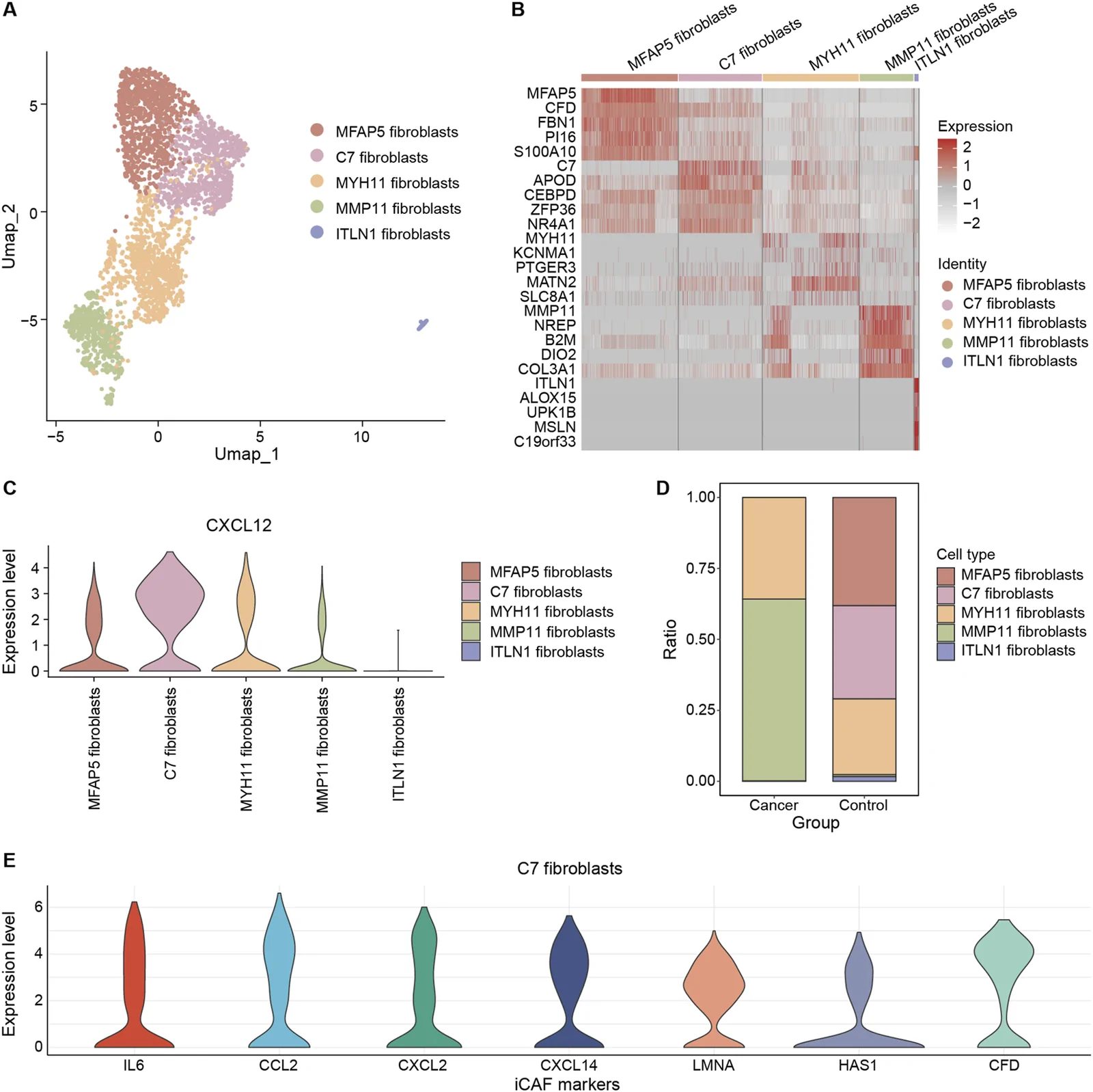
Single-cell transcriptomic landscape of fibroblasts. **(A)** Uniform manifold approximation and projection (UMAP) visualization delineating five fibroblast subpopulations, with each cell type indicated by a distinct color; **(B)** Heatmap displaying the top five characteristic genes with high expression in each fibroblast subpopulation; **(C)** Violin plot displaying the expression levels of CXCL12 across different fibroblast subpopulations; **(D)** Cell proportion chart illustrating the percentage distribution of various fibroblast subpopulations in cervical cancer tissues (Cancer) and adjacent normal tissues (Control); **(E)** Violin plot displaying the expression levels of iCAF marker genes in the C7 fibroblast subpopulation.

Given the pivotal role of the CXCL12-ACKR3 axis in cervical cancer progression, we further analyzed the CXCL12 expression patterns across distinct fibroblast subtypes. Based on bioinformatics analysis of single-cell transcriptome sequencing data, all fibroblasts were divided into five subclusters. To screen the specific differentially expressed genes of each subcluster, each single fibroblast subcluster was set as the experimental group, and the remaining four fibroblast subclusters were integrally set as the control group for intergroup transcriptomic differential expression analysis. Genes with *P* < 0.05 and avg_log2FC > 0 were defined as specifically highly expressed genes of each subcluster. Specifically, *P* < 0.05 indicated that the gene expression level in the target subcluster was significantly higher than that in the other four subclusters, and avg_log2FC > 0 indicated that the average gene expression level was higher in the target subcluster. A higher avg_log2FC value represented a more significant upregulation of gene expression in the target subcluster. The gene with the highest avg_log2FC value among the characteristic highly expressed genes of each fibroblast subcluster was selected as the signature gene of the corresponding fibroblast subcluster, and the five fibroblast subclusters were named accordingly: MFAP5 fibroblasts, C7 fibroblasts, MYH11 fibroblasts, MMP11 fibroblasts, and ITLN1 fibroblasts (Figures 5A,B).

Further analysis revealed that CXCL12 was highly expressed in the C7 fibroblast subpopulation (Figure 5C). Cell proportion analysis indicated that this subpopulation was predominantly distributed within adjacent normal tissues (Figure 5D). These findings are consistent with our previous observations. In addition, the C7 fibroblast subcluster identified in our study is not an exclusive finding of our work. Other published single-cell transcriptomic studies have also identified C7-positive fibroblast subpopulations in other diseases, which further corroborates the objective existence of this cell subpopulation (Tan et al., 2022; Fonseca et al., 2023).

CXCL12 is a well-recognized marker of iCAFs (Elyada et al., 2019). In our analysis, C7 fibroblasts exhibited high CXC12 expression (Figure 5C). To further evaluate their identity, we examined additional established iCAF marker genes (Elyada et al., 2019; Chen et al., 2020; Öhlund et al., 2017), including IL6, CCL2, CXCL2, CXCL14, LMNA, HAS1, and CFD, which were also highly expressed in C7 fibroblasts (Figure 5E). Therefore, we infer that C7 fibroblasts represents a subpopulation of iCAFs.

### 3.4 C7 fibroblasts are associated with cervical cancer progression through multiple genes and signaling pathways

**FIGURE 6:**
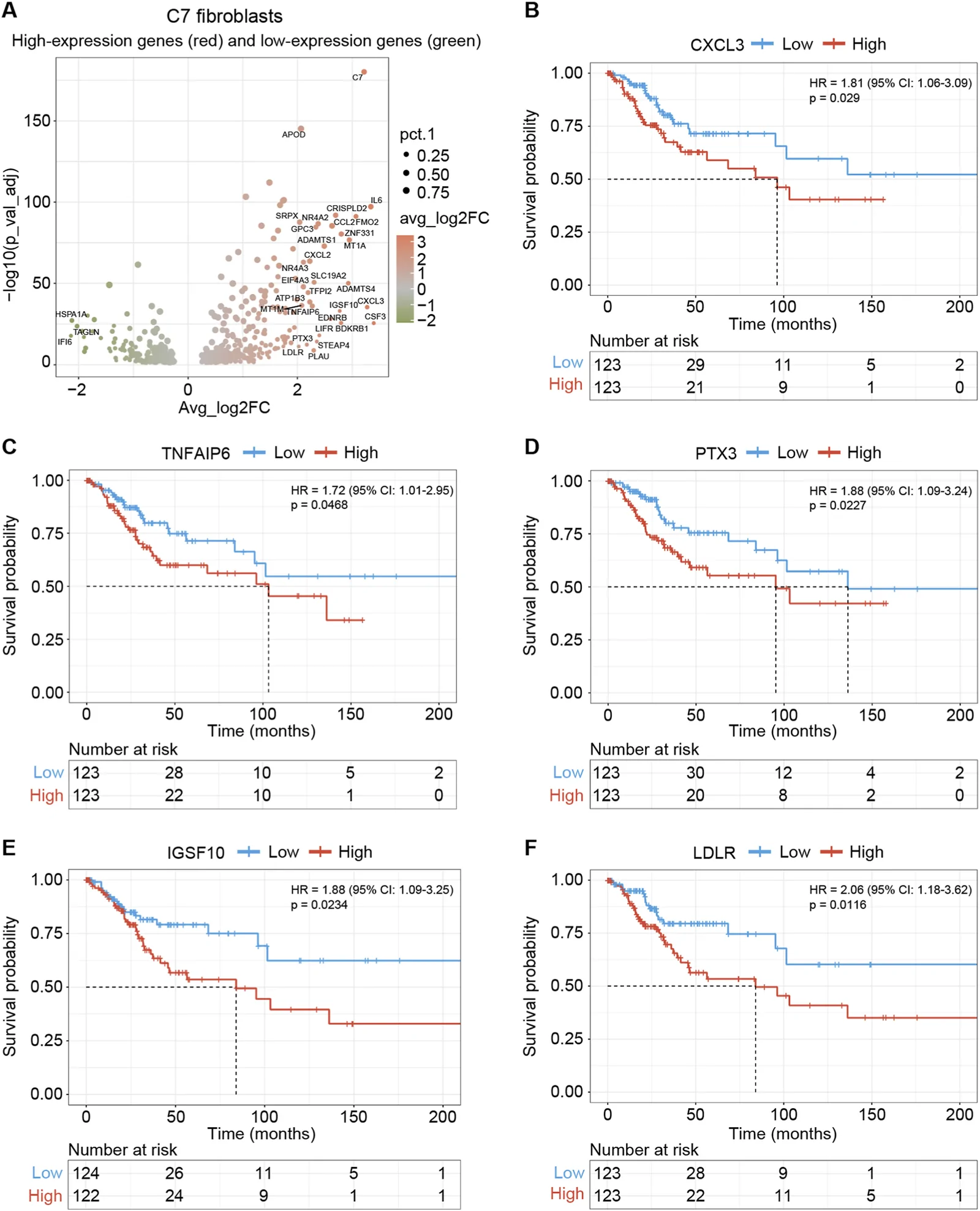
Significantly differentially expressed genes of iCAF subpopulation C7 fibroblasts and prognostic analysis based on The Cancer Genome Atlas Database. **(A)** Volcano plot illustrating the 463 significantly differentially expressed genes of iCAF subpopulation C7 fibroblasts with an adjusted p value (p_val_adj) < 0.05 and an absolute average log2 fold change (|avg_log2FC|) > 0. Due to the excessive number of genes in the plot, all gene names cannot be marked one by one. Therefore, we selectively labeled genes with p_val_adj <0.05 and |avg_log2FC| > 2; (b–f) Kaplan–Meier survival plots illustrating the associations between the expression levels of five candidate genes and overall survival in patients with cervical cancer, including CXCL3 **(B)**, TNFAIP6 **(C)**, PTX3 **(D)**, IGSF10 **(E)**, and LDLR **(F)**.

To further characterize the transcriptional profile of the iCAF subpopulation C7 fibroblasts and elucidate its role in cervical cancer, we analyzed the characteristic DEGs of this population. A total of 463 significant DEGs were identified (p_val_adj <0.05; |avg_log2FC| >0), including 277 upregulated and 186 downregulated genes (Figure 6A; Supplementary Table S4).

Among the genes that were significantly upregulated in this subpopulation, we identified several key factors that are closely associated with the initiation, progression, and prognosis of cervical cancer, including IL6, CXCL3, CXCL2, CCL2, GPC3, PLAU, TNFAIP6, PTX3, and EIF4A3 (Han et al., 2025; Walch-Rückheim et al., 2015; Wei et al., 2001; Qi et al., 2020; Zhang et al., 2018; Huang et al., 2020; Gao et al., 2024; Zhang et al., 2022; Hu and Zhu, 2019; Gao et al., 2022; Daneri-Navarro et al., 1998; Kobayashi et al., 1994; Hu et al., 2025; Ying et al., 2016; Yang et al., 2022), as well as CXCL3, TNFAIP6, PTX3, IGSF10, and LDLR, which are markedly linked to poor prognosis (Figures 6B–E; Supplementary Table S5). These factors are involved in multiple signaling pathways, including PI3K/Akt, JAK2/STAT3, and MAPK/ERK, and may cooperatively promote tumor cell proliferation, invasion, and angiogenesis, while contributing to remodeling of the immune microenvironment.

Taken together, these findings suggest that the iCAF subpopulation of C7 fibroblasts may participate in cervical cancer cell proliferation, invasion, angiogenesis, and immune microenvironment remodeling through multiple genes and signaling pathways, and that it is closely associated with cervical cancer progression.

### 3.5 Tumor-adjacent normal tissues have a higher abundance of C7 fibroblasts than cancer tissues

**FIGURE 7:**
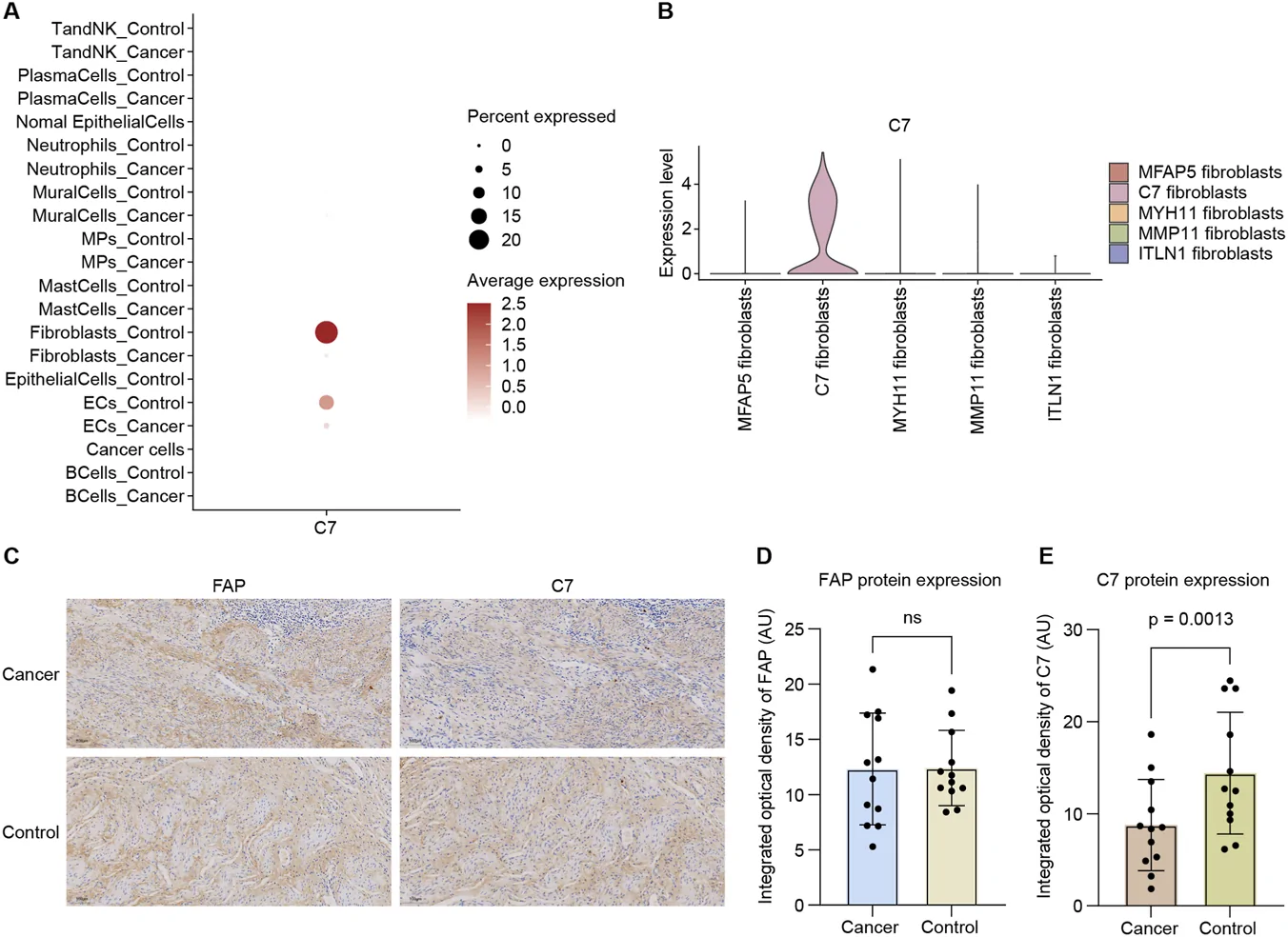
Expression of FAP and C7 in different cell types and clinical samples. **(A)** Bubble plot showing the expression levels of C7 across different cell types; **(B)** Violin plot showing the expression levels of C7 across fibroblast subpopulations; **(C)** Immunohistochemical images showing FAP expression in cancer tissues (Cancer) and the corresponding C7 expression in the same location on consecutive sections, as well as FAP expression in adjacent normal tissues (Control) and the corresponding C7 expression in the same location on consecutive sections; **(D)** Statistical plot showing FAP expression levels in FAP-positive regions of cancer tissues (Cancer) and adjacent normal tissues (Control) with comparable immunohistochemical staining intensity, along with the statistical differences between these groups; **(E)** Statistical plot showing C7 expression levels in the corresponding locations on consecutive sections of cancer tissues (Cancer) and adjacent normal tissues (Control) with a similar FAP immunohistochemical staining intensity, along with the statistical differences between these groups.

FAP is widely recognized as a representative marker of fibroblasts (Chen and Song, 2019; Kalluri, 2016; Bueno-Urquiza et al., 2024), while C7 is the highest expressed gene in C7 fibroblasts (Figure 6A). Therefore, we selected FAP and C7 as defining markers to investigate the presence of C7 fibroblasts. The results revealed that, across all cell types and different fibroblast subclusters, C7 expression was predominantly enriched in fibroblasts from adjacent normal tissues (Figures 7A,B). Based on these observations, and considering that this subpopulation is mainly distributed in adjacent normal tissues (Figure 5D), FAP and C7 were identified as specific markers of C7 fibroblasts.

Next, we subjected formalin-fixed paraffin-embedded (FFPE) tissue sections from cervical cancer lesions and their corresponding adjacent normal tissues (n = 12) to immunohistochemical staining (Supplementary Table S6). C7 expression was observed in FAP-positive regions in both cancerous and adjacent normal tissues (Figure 7C). Within regions exhibiting comparable FAP positivity (Figures 7C,D), the C7 expression levels in adjacent normal tissues were significantly higher than those in the corresponding cancer tissues (Figures 7C,E). Collectively, these findings demonstrate that C7 fibroblasts is more abundant in adjacent normal tissues than in cancer tissues.

### 3.6 The abundance of C7 fibroblasts in tumor-adjacent normal tissues is correlated with advanced FIGO stage, lymph node metastasis, and postmenopausal status

In the preceding analysis, we confirmed the high abundance of the iCAF subpopulation C7 fibroblasts in adjacent normal tissues. On the basis of these findings, we further investigated the association between the abundance of this cell population in adjacent normal tissues and the clinical features of patients with cervical cancer. To this end, we collected the following clinical information from 81 patients with cervical cancer: differentiation grade, FIGO stage, lymph node metastasis status, lymphovascular invasion status, perineural invasion status, abnormal uterine bleeding status, menopausal status, and HPV status (Supplementary Tables S7, S8).

**FIGURE 8:**
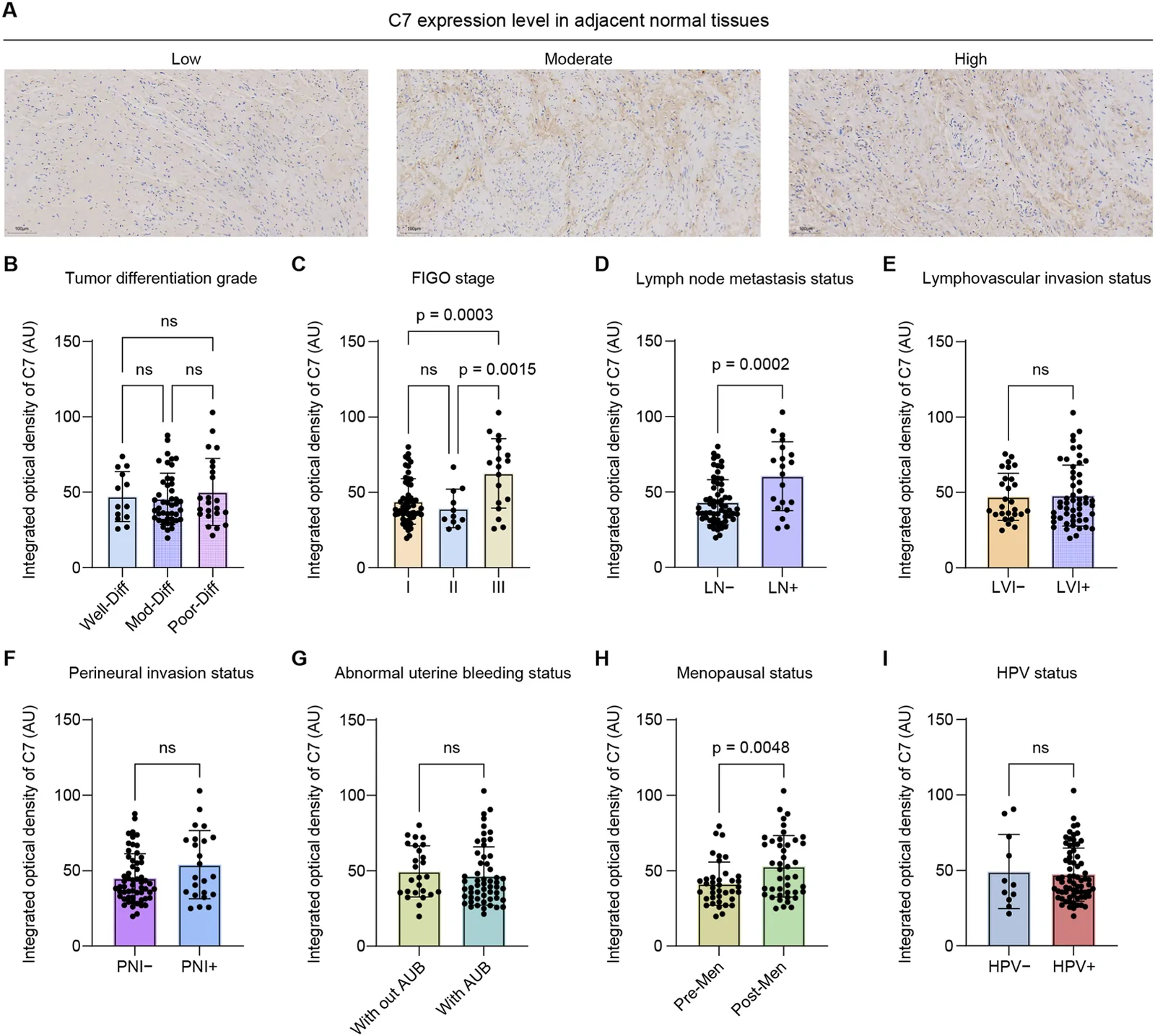
Association between C7 expression levels in tumor-adjacent normal tissues and clinicopathological features. **(A)** Representative immunohistochemical images showing variable C7 expression levels in adjacent normal tissues among patients with cervical cancer; **(B–I)** Statistical plots illustrating the associations between C7 expression levels in adjacent normal tissues and various clinicopathological parameters: **(B)** tumor differentiation grade, **(C)** International Federation of Gynecology and Obstetrics (FIGO) stage, **(D)** lymph node metastasis (LN) status, **(E)** lymphovascular invasion (LVI) status, **(F)** perineural invasion (PNI) status, **(G)** abnormal uterine bleeding (AUB) status, **(H)** menopausal status, and **(I)** human papillomavirus (HPV) status.

Immunohistochemistry was performed to evaluate the expression levels of C7 in adjacent normal tissues from 81 patients with cervical cancer. Considerable variability in C7 expression was observed among these patients (Figure 8A). Subsequently, the associations between various clinical characteristics and C7 expression levels in adjacent normal tissues were investigated (Figures 8B–I). The results demonstrated that patients with FIGO stage III exhibited significantly higher C7 expression in peri-tumoral adjacent tissues than those with FIGO stage I or II (Figure 8C). Similarly, patients with lymph node metastasis showed higher C7 expression in adjacent normal tissues than those without (Figure 8D). In addition, postmenopausal patients had higher C7 expression levels in adjacent normal tissues than premenopausal patients (Figure 8H).

Collectively, these data reveal that the iCAF subpopulation of C7 fibroblasts is closely associated with cervical cancer invasion and migration, providing further evidence for its pivotal role in the cervical cancer progression.

### 3.7 C7 fibroblasts are highly differentiated and exhibit transcriptional continuity with fibroblasts in cancer tissues

**FIGURE 9:**
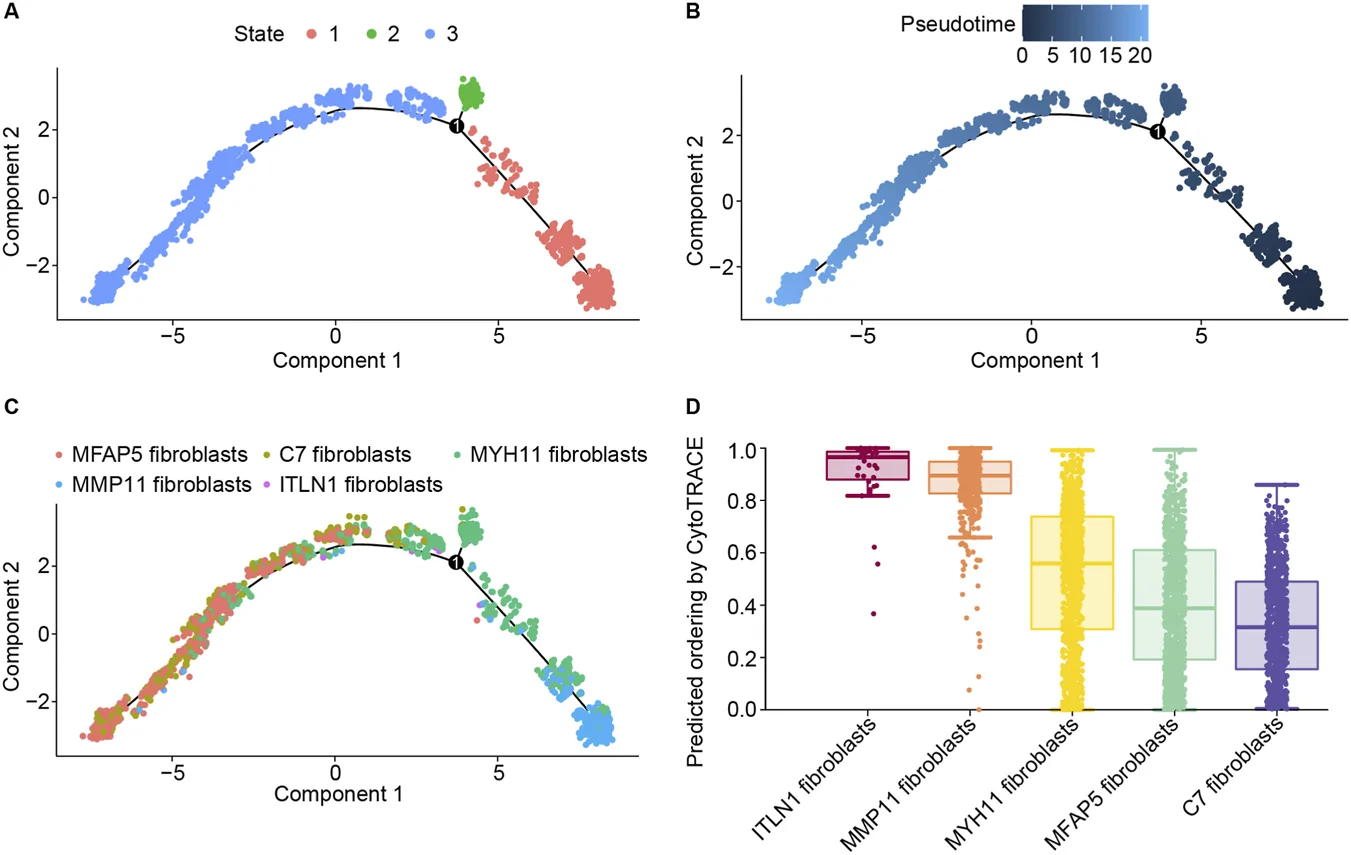
Fibroblast pseudotime analysis. **(A)** Cell state trajectory diagram showing fibroblasts classified into three states. **(B)** Pseudotime trajectory plot showing the fibroblast developmental trajectory (pseudotime 0–20). Colors transition from dark blue to light blue, indicating fibroblasts evolving from low to high differentiation. **(C)** Trajectory plot colored by cell type, showing the distribution of different fibroblast subtypes along the developmental trajectory. **(D)** CytoTRACE score plot for different fibroblast subtypes. Higher scores indicate lower differentiation levels.

We confirmed that C7 fibroblasts play a crucial role in promoting cervical cancer progression. To explore the origin and differentiation of these fibroblasts, we conducted pseudotime analysis to assess the differentiation degree and transcriptional continuity of fibroblasts. The analysis revealed that fibroblasts can be divided into three states: state1, state2, and state3 (Figure 9A). The pseudotime trajectory diagram corresponding to fibroblast cell states showed a developmental progression ranging from pseudotime 0 to 20. The color gradually changed from dark blue to light blue, representing the evolution of fibroblasts from low differentiation to high differentiation states (Figure 9B). Combined analysis of the two graphs demonstrated that fibroblasts in State 1 were located at the initial stage of the pseudotime trajectory and displayed a dark blue color, indicating a low differentiation level. Fibroblasts in State 2 were positioned in the intermediate stage of the pseudotime trajectory, with colors ranging between dark blue and light blue, suggesting that these cells were in a transitional differentiation state. Fibroblasts in State 3 were located at the later stage of the pseudotime trajectory and exhibited a light blue color, indicating a high differentiation level.

Subsequently, we mapped different fibroblast subtypes onto cell states and pseudotime developmental trajectories (Figure 9C). We found that MMP11 fibroblasts were located at the starting point of the trajectory state1, indicating a low differentiation status (Figure 9C). MYH11 fibroblasts appeared in state1, state2, and state3, suggesting they were in a transitional differentiation state (Figure 9C). C7 and MFAP5 fibroblasts were positioned in state3, indicating their high differentiation (Figure 9C). Due to the low number of ITLN1 fibroblasts, they are not discussed in this study. To further validate these findings, we analyzed CytoTRACE scores across fibroblast subtypes. CytoTRACE scores were negatively correlated with cellular differentiation, with higher scores indicating lower differentiation (Figure 9D). The results showed that MMP11 fibroblasts had the highest CytoTRACE scores, followed by MYH11 fibroblasts, whereas C7 and MFAP5 fibroblasts exhibited lower scores. These results are consistent with the pseudotime analysis, further supporting and validating our conclusions.

Additionally, combined with the distribution patterns of different fibroblast subtypes in cancer tissues and tumor-adjacent normal tissues (Figure 5D), we found that MMP11 fibroblasts with low differentiation levels were predominantly distributed in cancer tissues. MYH11 fibroblasts in a transitional differentiation state were distributed across both tissue types, while C7 fibroblasts with high differentiation levels were primarily present in tumor-adjacent normal tissues. Based on the general developmental progression from low differentiation to high differentiation states, C7 fibroblasts appear to exhibit transcriptional continuity with fibroblasts in cancer tissues and may evolve from low-differentiation fibroblasts within cancer tissues.

In conclusion, those C7 fibroblasts predominantly distributed in tumor-adjacent normal tissues exhibit relatively high differentiation levels and transcriptional continuity with low-differentiation fibroblasts in cancer tissues.

## 4 Discussion

Cervical cancer is one of the most prevalent gynecological malignancies, predominantly affecting populations in low- and middle-income countries, and represents a significant global public health burden (Lin et al., 2025; Han et al., 2024). The TME comprises diverse cellular components and their secreted cytokines, which interact dynamically to collectively influence tumor progression (Hanahan, 2022). Among these components, CAFs are widely recognized as key regulators or orchestrators of tumorigenesis. They play a critical role in the progression of various cancers, including cervical cancer, by modulating essential processes such as tumor cell proliferation, migration, invasion, metastasis, and resistance to therapy (Bueno-Urquiza et al., 2024; Wright et al., 2023; Hu et al., 2019; Richards et al., 2017).

CAFs play a key role in various stages of cervical cancer progression. On one hand, CAFs secrete multiple pro-cancer factors such as TGF-β1, SDF-1 CXCL12, Periostin, CCL20, and HB-EGF. These factors enhance cervical cancer cell proliferation, migration, invasion, and distant metastasis by promoting epithelial-mesenchymal transition, mediating extracellular matrix remodeling, inducing tumor angiogenesis, and disrupting the endothelial barrier (Wei et al., 2023; Bueno-Urquiza et al., 2024; Richards et al., 2017; Murata et al., 2011; Nurmik et al., 2020; Xiao et al., 2022). On the other hand, CAFs shape the immunosuppressive microenvironment by secreting chemokines. They secrete CCL2 to drive macrophage reprogramming and polarization towards the M2 phenotype, while also secreting CCL20, a Th17 chemokine, to promote the recruitment of Th17 cells to the tumor tissue (Sheng et al., 2023; Theobald et al., 2021). In addition, CAFs inhibit the infiltration of CD8^+^ T cells into the tumor tissue, assisting tumor cells in evading anti-tumor immune surveillance, thereby further promoting the formation and maintenance of the immunosuppressive microenvironment (Chen et al., 2025; Guo et al., 2024).

In this study, we systematically analyzed the cell-cell interactions between fibroblasts and cancer cells in both cancer and tumor-adjacent normal tissues. The results revealed that the tumor-adjacent iCAF subpopulation C7 fibroblasts plays a crucial role in promoting the progression of cervical cancer. We found that this subpopulation secreted the ligand CXCL12, which binds to the ACKR3 (CXCR7) receptor expressed on cancer cells, thereby markedly enhancing the proliferation, invasion, and metastasis of cervical cancer cells (Xu et al., 2021).

Among the signature highly expressed genes in this cell subpopulation, we identified several that can promote the occurrence and development of cervical cancer, including IL6, CXCL3, CXCL2, CCL2, GPC3, PLAU, TNFAIP6, PTX3, and EIF4A3. Among these, CXCL3, TNFAIP6, PTX3, IGSF10, and LDLR were associated with poor prognosis in cervical cancer. Immunohistochemical analysis of clinical specimens showed that high expression of the iCAF subpopulation C7 fibroblasts in tumor-adjacent tissues was significantly correlated with higher FIGO stage, lymph node metastasis, and postmenopausal status in patients with cervical cancer. Furthermore, we found that C7 fibroblasts were highly differentiated and exhibited transcriptional continuity with fibroblasts in cancer tissues.

Our results show that not only CAFs in cancer tissues promote cervical cancer progression, but CAFs in tumor-adjacent normal tissues also play a crucial role in tumor progression through specific molecular mechanisms. C7 fibroblasts in the tumor-adjacent normal tissue secrete various factors that regulate the tumor microenvironment, promoting cancer cell proliferation, invasion, and metastasis. This finding highlights the tumor-adjacent normal tissue as a potentially key environment for tumor development, providing support for the cancer tissue as well as a favorable biological context for tumor progression.

Although the present study provides novel insights into the role of fibroblasts in tumor-adjacent normal tissue during cervical cancer progression, several limitations should be acknowledged. First, the single-cell sequencing samples and clinical tissue sections in this study only included cervical squamous cell carcinoma, and they did not cover other pathological subtypes. Given that different histological subtypes of cervical cancer vary in biological features and clinical manifestations, our conclusions may not be generalizable across all subtypes. Further studies including additional cervical cancer subtypes are warranted to validate the presence and function of C7 fibroblasts in diverse histological contexts. Second, this study only preliminarily explored the function of C7 fibroblasts in cervical cancer progression at the bioinformatics level. As no specific cell surface markers for this subset have yet been identified, we were unable to isolate C7 fibroblasts from tissue samples using flow cytometry or other methods for further *in vitro* and *in vivo* functional verification. Future work will focus on identifying specific surface markers of C7 fibroblasts to enable cell sorting and subsequent *in vitro* cellular assays, as well as animal experiments. Third, although this study revealed the key role of C7 fibroblasts in tumor-adjacent normal tissue in cervical cancer progression, we analyzed their crosstalk with cancer cells and did not examine their interactions with other components of the tumor microenvironment. Future research should investigate the interactions between C7 fibroblasts and other tumor microenvironment components such as immune cells and vascular endothelial cells, to further clarify their effects on tumor growth, metastasis, and immune escape. Fourth, although the pseudotime analysis in this study suggests that C7 fibroblasts exhibit transcriptional continuity with fibroblasts in cancer tissue, the actual developmental relationship between these cells cannot be fully clarified through bioinformatics analysis alone. Further studies using lineage tracing and spatial omics approaches are required to accurately verify and further elucidate their developmental lineage and relationships. Finally, although immunohistochemical analysis showed that C7 fibroblasts were associated with higher FIGO stage, lymph node metastasis, and menopausal status in cervical cancer patients, these findings require validation in large-scale prospective cohorts. Multivariate analysis should be conducted to evaluate the association between C7 fibroblasts and other clinical features of cervical cancer, as well as their potential as clinical prognostic markers.

## 5 Conclusion

In summary, this study reveals the presence of a pro-tumorigenic iCAF subpopulation, C7 fibroblasts, within often-overlooked tumor-adjacent normal tissue. This subpopulation not only participates in cervical cancer progression through multiple signaling pathways at the molecular level, but it is also closely associated with higher FIGO stage, lymph node metastasis, and menopausal status at the clinical level. These findings suggest that tumor-adjacent normal tissue, as part of the tumor microenvironment, provides a supportive niche for tumor development and plays a critical role in cervical cancer progression. Additionally, our analysis indicates that C7 fibroblasts are highly differentiated and exhibit transcriptional continuity with fibroblasts in cancer tissues, highlighting the dynamic remodeling of CAFs in terms of spatial distribution and cellular origin within the tumor microenvironment. This study moves beyond the traditional paradigm focused solely on cancer tissue and underscores the important role of tumor-adjacent normal tissue in tumorigenesis and progression, thereby broadening the understanding of tumor microenvironment heterogeneity in cervical cancer. Our findings offer a new perspective on cervical cancer and suggest potential avenues for early prognostic assessment, staging optimization, and targeted therapeutic strategies.

## Data Availability

The datasets presented in this study can be found in online repositories. The names of the repository/repositories and accession number(s) can be found below: https://www.ncbi.nlm.nih.gov/, PRJNA1421171.
