# Supplementary material for: Single-cell transcriptomics reveals a correlation between inflammatory C7 fibroblasts in cervical tumor-adjacent normal tissues and cervical cancer progression
Source: Front Cell Dev Biol. 2026 Aug 10;14:1829843. doi: 10.3389/fcell.2026.1829843 (PMC13500651; doi:10.3389/fcell.2026.1829843)
Supplement: Supplementary file 1 [file Supplementaryfile1.docx]

Supplementary Material

**Supplementary Table 1** Lists 64 ligand–receptor interaction gene pairs between fibroblasts from cancer tissues and cancer cells.

| **Ligand** | **Receptor** |
| --- | --- |
| IGF1 | ITGA6_ITGB4 |
| MIF | ACKR3 |
| MDK | SDC1 |
| MDK | SDC4 |
| MDK | ITGA6_ITGB1 |
| MDK | LRP1 |
| MDK | NCL |
| PTN | SDC1 |
| PTN | SDC4 |
| PTN | NCL |
| COL1A1 | ITGA2_ITGB1 |
| COL1A2 | ITGA2_ITGB1 |
| COL4A1 | ITGA2_ITGB1 |
| COL4A2 | ITGA2_ITGB1 |
| COL6A1 | ITGA2_ITGB1 |
| COL6A2 | ITGA2_ITGB1 |
| COL6A3 | ITGA2_ITGB1 |
| LAMA2 | ITGA2_ITGB1 |
| LAMA4 | ITGA2_ITGB1 |
| LAMB1 | ITGA2_ITGB1 |
| LAMC1 | ITGA2_ITGB1 |
| LAMA2 | ITGA6_ITGB1 |
| LAMA4 | ITGA6_ITGB1 |
| LAMB1 | ITGA6_ITGB1 |
| LAMC1 | ITGA6_ITGB1 |
| LAMA2 | ITGA6_ITGB4 |
| LAMA4 | ITGA6_ITGB4 |
| LAMB1 | ITGA6_ITGB4 |
| LAMC1 | ITGA6_ITGB4 |
| FN1 | CD44 |
| COL1A1 | CD44 |
| COL1A2 | CD44 |
| COL4A1 | CD44 |
| COL4A2 | CD44 |
| COL6A1 | CD44 |
| COL6A2 | CD44 |
| COL6A3 | CD44 |
| LAMA2 | CD44 |
| LAMA4 | CD44 |
| LAMB1 | CD44 |
| LAMC1 | CD44 |
| COL1A1 | SDC1 |
| COL1A2 | SDC1 |
| COL4A1 | SDC1 |
| COL4A2 | SDC1 |
| COL6A1 | SDC1 |
| COL6A2 | SDC1 |
| COL6A3 | SDC1 |
| FN1 | SDC1 |
| THBS1 | SDC1 |
| THBS2 | SDC1 |
| COL1A1 | SDC4 |
| COL1A2 | SDC4 |
| COL4A1 | SDC4 |
| COL4A2 | SDC4 |
| COL6A1 | SDC4 |
| COL6A2 | SDC4 |
| COL6A3 | SDC4 |
| FN1 | SDC4 |
| THBS1 | SDC4 |
| THBS2 | SDC4 |
| THBS1 | CD47 |
| THBS2 | CD47 |
| CD99 | CD99 |

The ligands originate from fibroblasts within cancer tissues, while the receptors derive from cancer cells.

**Supplementary Table 2** Lists 46 ligand–receptor interaction gene pairs between fibroblasts from adjacent normal tissues and cancer cells.

| **Ligand** | **Receptor** |
| --- | --- |
| IGF1 | ITGA6_ITGB4 |
| CXCL12 | ACKR3 |
| COL1A1 | ITGA2_ITGB1 |
| COL1A2 | ITGA2_ITGB1 |
| COL6A1 | ITGA2_ITGB1 |
| COL6A2 | ITGA2_ITGB1 |
| COL6A3 | ITGA2_ITGB1 |
| LAMA2 | ITGA2_ITGB1 |
| LAMA4 | ITGA2_ITGB1 |
| LAMC1 | ITGA2_ITGB1 |
| LAMA2 | ITGA6_ITGB1 |
| LAMA4 | ITGA6_ITGB1 |
| LAMC1 | ITGA6_ITGB1 |
| LAMA2 | ITGA6_ITGB4 |
| LAMA4 | ITGA6_ITGB4 |
| LAMC1 | ITGA6_ITGB4 |
| FN1 | CD44 |
| COL1A1 | CD44 |
| COL1A2 | CD44 |
| COL6A1 | CD44 |
| COL6A2 | CD44 |
| COL6A3 | CD44 |
| LAMA2 | CD44 |
| LAMA4 | CD44 |
| LAMC1 | CD44 |
| COL1A1 | SDC1 |
| COL1A2 | SDC1 |
| COL6A1 | SDC1 |
| COL6A2 | SDC1 |
| COL6A3 | SDC1 |
| FN1 | SDC1 |
| TNXB | SDC1 |
| THBS1 | SDC1 |
| THBS2 | SDC1 |
| COL1A1 | SDC4 |
| COL1A2 | SDC4 |
| COL6A1 | SDC4 |
| COL6A2 | SDC4 |
| COL6A3 | SDC4 |
| FN1 | SDC4 |
| TNXB | SDC4 |
| THBS1 | SDC4 |
| THBS2 | SDC4 |
| THBS1 | CD47 |
| THBS2 | CD47 |
| CD99 | CD99 |

The ligands originate from fibroblasts within adjacent normal tissues, while the receptors derive from cancer cells.

**Supplementary Table 3** Unique ligand–receptor interaction gene pairs between Fibroblasts_Cancer or Fibroblasts_Control and cancer cells.

| Group | Fibroblasts_Cancer → Cancer cells  unique L-R gene pairs | Fibroblasts_Control → Cancer cells  unique L-R gene pairs |
| --- | --- | --- |
| Number | 21 | 3 |
| L–R gene pairs | MIF_ACKR3 | CXCL12_ACKR3 (CXCR7) |
|  | MDK_SDC1 / MDK_SDC4 / MDK_LRP1 | TNXB_SDC1 |
|  | MDK_ITGA6_ITGB1 / MDK_NCL | TNXB_SDC4 |
|  | PTN_SDC1 / PTN_SDC4 / PTN_NCL |  |
|  | COL4A1_ITGA2_ITGB1 / COL4A1_CD44 |  |
|  | COL4A2_ITGA2_ITGB1 / COL4A2_CD44 |  |
|  | COL4A1_SDC1 / COL4A1_SDC4 |  |
|  | COL4A2_SDC1 / COL4A2_SDC4 |  |

Fibroblasts_Cancer: Fibroblasts within cancer tissues; Fibroblasts_Control: Fibroblasts within umor-adjacent normal tissues.

**Supplementary Table 4** Significant Differentially Expressed Genes of the iCAFs Subpopulation Fibroblasts_C7.

| **Gene** | **Patterns** | **Gene** | **Patterns** |
| --- | --- | --- | --- |
| CSF3 | Up | LAPTM4A | Down |
| IL6 | Up | MT-CO2 | Down |
| CXCL3 | Up | RPL30 | Down |
| C7 | Up | RPL23A | Down |
| FMO2 | Up | RPL13A | Down |
| MT1A | Up | RPL31 | Down |
| ADAMTS4 | Up | MT-CO3 | Down |
| ZNF331 | Up | RPS25 | Down |
| BDKRB1 | Up | RPL23 | Down |
| IGSF10 | Up | RPL13 | Down |
| CRISPLD2 | Up | RPS16 | Down |
| CCL2 | Up | RPL8 | Down |
| EDNRB | Up | RPL27 | Down |
| ADAMTS1 | Up | TXN | Down |
| LIFR | Up | RPL12 | Down |
| NR4A2 | Up | RPS23 | Down |
| STEAP4 | Up | RPS13 | Down |
| GPC3 | Up | RPS2 | Down |
| SLC19A2 | Up | RPL29 | Down |
| PLAU | Up | RPLP1 | Down |
| TNFAIP6 | Up | RPL19 | Down |
| ATP1B3 | Up | RPL26 | Down |
| CXCL2 | Up | SRP14 | Down |
| TFPI2 | Up | DYNLL1 | Down |
| PTX3 | Up | CHCHD2 | Down |
| NR4A3 | Up | ATP6V0E1 | Down |
| EIF4A3 | Up | RPS14 | Down |
| MT1M | Up | RPL28 | Down |
| APOD | Up | RPS3A | Down |
| SRPX | Up | BTF3 | Down |
| LDLR | Up | RPS4X | Down |
| RGS2 | Up | RHOA | Down |
| ICAM1 | Up | RPL3 | Down |
| ELL2 | Up | PEBP1 | Down |
| CREM | Up | RPL5 | Down |
| PTGDS | Up | RPS12 | Down |
| RASD1 | Up | REXO2 | Down |
| KDM6B | Up | COX7A2 | Down |
| FST | Up | ACTG1 | Down |
| PPP1R15B | Up | RPS3 | Down |
| TIPARP | Up | RPS6 | Down |
| FSTL3 | Up | S100A11 | Down |
| REL | Up | CFL1 | Down |
| RANBP2 | Up | PRDX1 | Down |
| NR4A1 | Up | SKP1 | Down |
| NTRK2 | Up | RPL15 | Down |
| MYC | Up | EEF1A1 | Down |
| MIR22HG | Up | COX7C | Down |
| CXCL12 | Up | HLA-A | Down |
| FGL2 | Up | RAC1 | Down |
| EMP1 | Up | SOD1 | Down |
| ARID5A | Up | H3-3A | Down |
| NAMPT | Up | PPIA | Down |
| SVEP1 | Up | RPS27L | Down |
| PIM1 | Up | TMEM14C | Down |
| PIM3 | Up | NDUFB4 | Down |
| NFIL3 | Up | PTMA | Down |
| MT1X | Up | TUBB | Down |
| MMP19 | Up | TMSB10 | Down |
| CXCL14 | Up | EIF4A2 | Down |
| ADH1B | Up | CSDE1 | Down |
| THBS1 | Up | CNN3 | Down |
| MAFF | Up | TAX1BP3 | Down |
| MAT2A | Up | COX4I1 | Down |
| CEBPD | Up | ACTB | Down |
| ZC3H12A | Up | BANF1 | Down |
| CFI | Up | SEPTIN2 | Down |
| DPT | Up | IFITM3 | Down |
| PTGER4 | Up | SUMO2 | Down |
| NFKB1 | Up | ESD | Down |
| MT2A | Up | SNRPD2 | Down |
| SLIT3 | Up | ATP5MG | Down |
| ANGPTL1 | Up | BRI3 | Down |
| ABCA10 | Up | NDUFC1 | Down |
| NNMT | Up | AC010197.2 | Down |
| SOD2 | Up | ATP5F1C | Down |
| PLAUR | Up | FTH1 | Down |
| PTGS2 | Up | SERF2 | Down |
| C16orf89 | Up | ST13 | Down |
| SRGN | Up | MYL12B | Down |
| MSX1 | Up | RAB2A | Down |
| COLEC12 | Up | TRMT112 | Down |
| KLF9 | Up | PFDN5 | Down |
| FOSB | Up | NBL1 | Down |
| CDKN1A | Up | DAD1 | Down |
| HLA-DRB1 | Up | NAP1L1 | Down |
| BCL3 | Up | RPL27A | Down |
| SPON1 | Up | LGALS1 | Down |
| RHOU | Up | RBX1 | Down |
| HSPB6 | Up | NDUFA4 | Down |
| ENPEP | Up | OST4 | Down |
| GPRC5A | Up | COX6B1 | Down |
| PNRC1 | Up | COX6C | Down |
| TNFAIP2 | Up | COX7B | Down |
| COL15A1 | Up | CD151 | Down |
| PDK4 | Up | PSMB5 | Down |
| CYCS | Up | OSTC | Down |
| GEM | Up | MT-CO1 | Down |
| PLK2 | Up | ATP5MC3 | Down |
| F3 | Up | BRK1 | Down |
| BTG2 | Up | H1-0 | Down |
| PXDC1 | Up | ADI1 | Down |
| ZBTB21 | Up | CALM2 | Down |
| TMEM176A | Up | HNRNPA1 | Down |
| CEBPB | Up | COX8A | Down |
| XBP1 | Up | PALLD | Down |
| LMNA | Up | ARPC2 | Down |
| TNNT3 | Up | HLA-C | Down |
| AC087521.3 | Up | PARK7 | Down |
| CCNH | Up | MIF | Down |
| NRP1 | Up | UQCRQ | Down |
| OGFRL1 | Up | H1-10 | Down |
| LINC00632 | Up | PRSS23 | Down |
| NFATC1 | Up | VCAN | Down |
| CHD1 | Up | H4C3 | Down |
| RARRES1 | Up | MRPL33 | Down |
| CSRNP1 | Up | MYL6 | Down |
| PER1 | Up | UQCR10 | Down |
| IRF1 | Up | COPS9 | Down |
| INSIG1 | Up | PSMB3 | Down |
| SNHG9 | Up | ITGB5 | Down |
| SPSB1 | Up | ASPN | Down |
| C11orf96 | Up | ANAPC11 | Down |
| PLPP3 | Up | MFAP2 | Down |
| CDC42EP4 | Up | LAMTOR1 | Down |
| PDLIM3 | Up | MARCKS | Down |
| COL14A1 | Up | VDAC2 | Down |
| NFKBIA | Up | HLA-B | Down |
| FOSL2 | Up | CMTM3 | Down |
| ZFP36 | Up | ATP5MC2 | Down |
| FXYD1 | Up | TMSB4X | Down |
| ZFAND5 | Up | C19orf53 | Down |
| PER2 | Up | FLNA | Down |
| SOCS3 | Up | IFITM1 | Down |
| GFRA1 | Up | PKM | Down |
| PCF11 | Up | ARPC5 | Down |
| TMEM176B | Up | TPM2 | Down |
| ABCA8 | Up | GNG5 | Down |
| CCNL1 | Up | CAPZB | Down |
| SERPINF1 | Up | AC005747.1 | Down |
| KLF4 | Up | VMP1 | Down |
| CXCL8 | Up | CLTA | Down |
| WNT11 | Up | CTSB | Down |
| MLF1 | Up | HINT1 | Down |
| NOLC1 | Up | SPARC | Down |
| SLC43A3 | Up | CCN2 | Down |
| SLC2A3 | Up | GJA1 | Down |
| SPRY2 | Up | C4orf3 | Down |
| B4GALT1 | Up | PSME2 | Down |
| ABCA9 | Up | COL1A1 | Down |
| FGF7 | Up | GSTO1 | Down |
| PTPRS | Up | ZEB1 | Down |
| C3 | Up | GAPDH | Down |
| DDX21 | Up | YWHAH | Down |
| MYADM | Up | CWC15 | Down |
| BAG3 | Up | HSPB1 | Down |
| CRABP2 | Up | CD248 | Down |
| SERTAD1 | Up | MYH9 | Down |
| GAS6 | Up | MYL12A | Down |
| FAM13A | Up | COL5A2 | Down |
| PTGFR | Up | MMP14 | Down |
| EFEMP1 | Up | TCEAL4 | Down |
| RNASE4 | Up | MYL9 | Down |
| ETS2 | Up | OSR2 | Down |
| ABCA6 | Up | DYNLT1 | Down |
| MARCHF3 | Up | GBP2 | Down |
| EPHX1 | Up | ACKR3 | Down |
| MIDN | Up | HSPA1B | Down |
| ANK2 | Up | SPATS2L | Down |
| SAMHD1 | Up | CD55 | Down |
| OLFML3 | Up | SOX4 | Down |
| CTSL | Up | FN1 | Down |
| MGP | Up | ANTXR1 | Down |
| PCOLCE | Up | BGN | Down |
| JUND | Up | B2M | Down |
| PPL | Up | IFI27 | Down |
| SPRY1 | Up | COL3A1 | Down |
| RSPO3 | Up | PAM | Down |
| CYGB | Up | TPM1 | Down |
| SPART | Up | FBN1 | Down |
| ERRFI1 | Up | MFAP5 | Down |
| PLAGL1 | Up | SULF1 | Down |
| CPVL | Up | SFRP4 | Down |
| CD302 | Up | TAGLN | Down |
| FILIP1L | Up | HSPA1A | Down |
| GPX3 | Up | IFI6 | Down |
| JUNB | Up |  |  |
| SGCE | Up |  |  |
| KLF2 | Up |  |  |
| OMD | Up |  |  |
| ITIH5 | Up |  |  |
| CFH | Up |  |  |
| MEDAG | Up |  |  |
| ADAM33 | Up |  |  |
| LAMC1 | Up |  |  |
| SFRP2 | Up |  |  |
| MCL1 | Up |  |  |
| ARID5B | Up |  |  |
| CTSF | Up |  |  |
| PLBD1 | Up |  |  |
| DDX5 | Up |  |  |
| ANGPTL2 | Up |  |  |
| PODN | Up |  |  |
| SRSF3 | Up |  |  |
| C1R | Up |  |  |
| NFE2L2 | Up |  |  |
| SFRP1 | Up |  |  |
| H19 | Up |  |  |
| STAT3 | Up |  |  |
| DDX3X | Up |  |  |
| EGR1 | Up |  |  |
| ZFP36L2 | Up |  |  |
| SRRM2 | Up |  |  |
| GFPT2 | Up |  |  |
| HAS1 | Up |  |  |
| EMP2 | Up |  |  |
| OGN | Up |  |  |
| ATF3 | Up |  |  |
| YBX3 | Up |  |  |
| DCN | Up |  |  |
| NFIB | Up |  |  |
| FBLN1 | Up |  |  |
| DNAJA1 | Up |  |  |
| IGFBP7 | Up |  |  |
| PLXDC1 | Up |  |  |
| NR3C1 | Up |  |  |
| TFPI | Up |  |  |
| SCPEP1 | Up |  |  |
| ALDH1A1 | Up |  |  |
| SFPQ | Up |  |  |
| VIM | Up |  |  |
| LSP1 | Up |  |  |
| PRNP | Up |  |  |
| KLF6 | Up |  |  |
| SELENOP | Up |  |  |
| EIF1B | Up |  |  |
| DCLK1 | Up |  |  |
| CCDC80 | Up |  |  |
| HLA-E | Up |  |  |
| LAMA2 | Up |  |  |
| EIF1 | Up |  |  |
| CCN1 | Up |  |  |
| ID2 | Up |  |  |
| PLTP | Up |  |  |
| LRP1 | Up |  |  |
| MGST1 | Up |  |  |
| PPP1R15A | Up |  |  |
| WSB1 | Up |  |  |
| C1S | Up |  |  |
| GSN | Up |  |  |
| PID1 | Up |  |  |
| UBC | Up |  |  |
| CCN5 | Up |  |  |
| YWHAZ | Up |  |  |
| NEGR1 | Up |  |  |
| IGFBP4 | Up |  |  |
| GPNMB | Up |  |  |
| SOD3 | Up |  |  |
| ABI3BP | Up |  |  |
| FBLN5 | Up |  |  |
| IGFBP5 | Up |  |  |
| METTL7A | Up |  |  |
| ITM2A | Up |  |  |
| TIMP3 | Up |  |  |
| ADIRF | Up |  |  |
| MT-ATP8 | Up |  |  |
| SPTBN1 | Up |  |  |
| SPARCL1 | Up |  |  |
| DUSP1 | Up |  |  |
| FOS | Up |  |  |
| CYBRD1 | Up |  |  |
| COL6A2 | Up |  |  |
| PDGFRL | Up |  |  |
| SERPING1 | Up |  |  |
| CPE | Up |  |  |
| DYRK3 | Up |  |  |
| FOSL1 | Up |  |  |

**Supplementary Table 5** Mechanisms and prognostic implications of oncogenic genes in iCAF subpopulation C7 fibroblasts.

| Gene | **Mechanistic roles in cervical cancer** | **References/Figure** |
| --- | --- | --- |
| IL6 | Activates the PI3K/Akt and JAK2/STAT3 signaling pathways, thereby promoting the proliferation, invasion, and migration of cancer cells; induces CCL20 recruitment of Th17 cells via C/EBPβ, sustaining pro-tumor inflammation and angiogenesis. | [28–30] |
| CXCL3 | Activates the MAPK/ERK signaling pathway, thereby enhancing tumorigenic potential; this is associated with poor prognosis. | [31]; (Fig. 6b) |
| CXCL2 | Promotes angiogenesis and enhances cellular proliferation and survival capacity. | [32] |
| CCL2 | Activates the CCL2–CCR2 axis, promoting perineural invasion in cervical cancer and inducing epithelial–mesenchymal transition (EMT). | [33–35] |
| GPC3 | Promotes tumor proliferation and migration via the GPC3-AS1/GPC3 axis and regulates EMT processes. | [36] |
| PLAU | Degrades the extracellular matrix, thereby facilitating the proliferation, migration, and invasion of cancer cells. | [37–39] |
| TNFAIP6 | High expression is closely associated with radiotherapy resistance and poor prognosis. | [40]; (Fig. 6c) |
| PTX3 | Promotes proliferation, migration, and invasion; high expression is correlated with higher tumor grade, poor differentiation, and unfavorable prognosis. | [41]; (Fig. 6d) |
| EIF4A3 | Suppresses the circ_0087429–miR-5003-3p–OGN-mediated tumor-suppressive pathway, thereby facilitating EMT and tumor progression. | [42] |
| IGSF10 | High expression is associated with poor prognosis. | (Fig. 6e) |
| LDLR | High expression is associated with poor prognosis. | (Fig. 6f) |

**Supplementary Table 6** Clinical characteristics of 12 cervical cancer samples used for immunohistochemical analysis of FAP and C7 expression.

| **Sample ID** | **Age** | **Pathological type** | **Tumor differentiation grade** |
| --- | --- | --- | --- |
| Sample 1 | 38 | Cervical squamous cell carcinoma | Moderately differentiated |
| Sample 2 | 43 | Cervical squamous cell carcinoma | Moderately differentiated |
| Sample 3 | 57 | Cervical squamous cell carcinoma | Well-differentiated |
| Sample 4 | 52 | Cervical squamous cell carcinoma | Poorly differentiated |
| Sample 5 | 66 | Cervical squamous cell carcinoma | Moderately differentiated |
| Sample 6 | 53 | Cervical squamous cell carcinoma | Poorly differentiated |
| Sample 7 | 59 | Cervical squamous cell carcinoma | Well-differentiated |
| Sample 8 | 69 | Cervical squamous cell carcinoma | Poorly differentiated |
| Sample 9 | 54 | Cervical squamous cell carcinoma | Well-differentiated |
| Sample 10 | 54 | Cervical squamous cell carcinoma | Moderately differentiated |
| Sample 11 | 47 | Cervical squamous cell carcinoma | Poorly differentiated |
| Sample 12 | 45 | Cervical squamous cell carcinoma | Well-differentiated |

**Supplementary Table 7** Clinical characteristics of patients with cervical cancer (n = 81).

| **Characteristic/Category** | **n** | **%** |
| --- | --- | --- |
| **Tumor differentiation grade** |  |  |
| Well-differentiated | 14 | 17.28 |
| Moderately differentiated | 44 | 54.32 |
| Poorly differentiated | 23 | 28.40 |
| **FIGO stage** |  |  |
| Stage I | 52 | 64.20 |
| Stage II | 11 | 13.58 |
| Stage III | 18 | 22.22 |
| **Lymph node metastasis status** |  |  |
| LN− | 61 | 75.31 |
| LN+ | 20 | 24.69 |
| **Lymphovascular invasion status** |  |  |
| LVI− | 27 | 33.33 |
| LVI+ | 53 | 65.44 |
| NA | 1 | 1.23 |
| **Perineural invasion status** |  |  |
| PNI− | 59 | 72.84 |
| PNI+ | 22 | 27.16 |
| **Abnormal uterine bleeding status** |  |  |
| Without AUB | 26 | 32.10 |
| With AUB | 55 | 67.90 |
| **Menopausal status** |  |  |
| Premenopausal | 38 | 46.91 |
| Postmenopausal | 43 | 53.09 |
| **Human papillomavirus status** |  |  |
| HPV− | 11 | 13.58 |
| HPV+ | 70 | 86.42 |

NA: missing data. Among the 81 patients, 11 were not infected with HPV. This was intentionally selected because we aimed to investigate whether the abundance of C7 fibroblasts in tumor-adjacent normal tissue is associated with HPV infection.

**Supplementary Table 8** Clinical characteristics of patients with cervical cancer (n=81).

| **Patient ID** | **Age** | **Pathological type** | **Tumor differentiation grade** | **FIGO stage** | **Lymph node metastasis status** | **Lymphovascular invasion status** | **Perineural invasion status** | **Abnormal uterine bleeding status** | **Menopausal status** | **HPV status** |
| --- | --- | --- | --- | --- | --- | --- | --- | --- | --- | --- |
| Patient 1 | 32 | Cervical squamous cell carcinoma | Moderately differentiated | IB3 | LN− | LVI+ | PNI+ | Without AUB | Premenopausal | HPV− |
| Patient 2 | 44 | Cervical squamous cell carcinoma | Poorly differentiated | IB1 | LN− | LVI+ | PNI+ | With AUB | Premenopausal | HPV− |
| Patient 3 | 43 | Cervical squamous cell carcinoma | Poorly differentiated | IB2 | LN− | LVI+ | PNI− | With AUB | Premenopausal | HPV− |
| Patient 4 | 46 | Cervical squamous cell carcinoma | Poorly differentiated | IIA1 | LN+ | LVI+ | PNI+ | With AUB | Premenopausal | HPV− |
| Patient 5 | 66 | Cervical squamous cell carcinoma | Moderately differentiated | IB2 | LN− | LVI+ | PNI− | Without AUB | Postmenopausal | HPV− |
| Patient 6 | 54 | Cervical squamous cell carcinoma | Poorly differentiated | IIA1 | LN− | LVI+ | PNI+ | Without AUB | Postmenopausal | HPV− |
| Patient 7 | 58 | Cervical squamous cell carcinoma | Poorly differentiated | IB1 | LN− | LVI+ | PNI− | With AUB | Postmenopausal | HPV− |
| Patient 8 | 57 | Cervical squamous cell carcinoma | Well-differentiated | IB3 | LN− | LVI+ | PNI+ | With AUB | Postmenopausal | HPV− |
| Patient 9 | 63 | Cervical squamous cell carcinoma | Moderately differentiated | IIA1 | LN− | LVI+ | PNI− | With AUB | Postmenopausal | HPV− |
| Patient 10 | 59 | Cervical squamous cell carcinoma | Poorly differentiated | IIIC1 | LN+ | LVI+ | PNI+ | With AUB | Postmenopausal | HPV− |
| Patient 11 | 60 | Cervical squamous cell carcinoma | Moderately differentiated | IIIC1 | LN+ | LVI+ | PNI− | With AUB | Postmenopausal | HPV− |
| Patient 12 | 42 | Cervical squamous cell carcinoma | Moderately differentiated | IIIC1 | LN+ | NA | PNI− | Without AUB | Premenopausal | HPV+ |
| Patient 13 | 36 | Cervical squamous cell carcinoma | Well-differentiated | IA1 | LN− | LVI− | PNI− | Without AUB | Premenopausal | HPV+ |
| Patient 14 | 39 | Cervical squamous cell carcinoma | Moderately differentiated | IA1 | LN− | LVI− | PNI− | Without AUB | Premenopausal | HPV+ |
| Patient 15 | 46 | Cervical squamous cell carcinoma | Moderately differentiated | IA2 | LN− | LVI− | PNI− | Without AUB | Premenopausal | HPV+ |
| Patient 16 | 45 | Cervical squamous cell carcinoma | Well-differentiated | IB1 | LN− | LVI− | PNI− | Without AUB | Premenopausal | HPV+ |
| Patient 17 | 35 | Cervical squamous cell carcinoma | Moderately differentiated | IB1 | LN− | LVI− | PNI− | Without AUB | Premenopausal | HPV+ |
| Patient 18 | 25 | Cervical squamous cell carcinoma | Poorly differentiated | IB2 | LN− | LVI− | PNI− | Without AUB | Premenopausal | HPV+ |
| Patient 19 | 43 | Cervical squamous cell carcinoma | Moderately differentiated | IB1 | LN− | LVI+ | PNI− | Without AUB | Premenopausal | HPV+ |
| Patient 20 | 42 | Cervical squamous cell carcinoma | Poorly differentiated | IB2 | LN− | LVI+ | PNI− | Without AUB | Premenopausal | HPV+ |
| Patient 21 | 50 | Cervical squamous cell carcinoma | Moderately differentiated | IB2 | LN− | LVI+ | PNI− | Without AUB | Premenopausal | HPV+ |
| Patient 22 | 54 | Cervical squamous cell carcinoma | Well-differentiated | IB1 | LN− | LVI− | PNI− | With AUB | Premenopausal | HPV+ |
| Patient 23 | 47 | Cervical squamous cell carcinoma | Poorly differentiated | IB2 | LN− | LVI− | PNI− | With AUB | Premenopausal | HPV+ |
| Patient 24 | 42 | Cervical squamous cell carcinoma | Moderately differentiated | IB3 | LN− | LVI− | PNI− | With AUB | Premenopausal | HPV+ |
| Patient 25 | 54 | Cervical squamous cell carcinoma | Poorly differentiated | IB1 | LN− | LVI+ | PNI− | With AUB | Premenopausal | HPV+ |
| Patient 26 | 40 | Cervical squamous cell carcinoma | Moderately differentiated | IB1 | LN− | LVI+ | PNI− | With AUB | Premenopausal | HPV+ |
| Patient 27 | 44 | Cervical squamous cell carcinoma | Moderately differentiated | IB1 | LN− | LVI+ | PNI− | With AUB | Premenopausal | HPV+ |
| Patient 28 | 49 | Cervical squamous cell carcinoma | Moderately differentiated | IB1 | LN− | LVI+ | PNI− | With AUB | Premenopausal | HPV+ |
| Patient 29 | 52 | Cervical squamous cell carcinoma | Poorly differentiated | IB2 | LN− | LVI+ | PNI− | With AUB | Premenopausal | HPV+ |
| Patient 30 | 53 | Cervical squamous cell carcinoma | Poorly differentiated | IB2 | LN− | LVI+ | PNI− | With AUB | Premenopausal | HPV+ |
| Patient 31 | 38 | Cervical squamous cell carcinoma | Moderately differentiated | IB2 | LN− | LVI+ | PNI− | With AUB | Premenopausal | HPV+ |
| Patient 32 | 39 | Cervical squamous cell carcinoma | Moderately differentiated | IB2 | LN− | LVI+ | PNI− | With AUB | Premenopausal | HPV+ |
| Patient 33 | 40 | Cervical squamous cell carcinoma | Moderately differentiated | IB2 | LN− | LVI+ | PNI− | With AUB | Premenopausal | HPV+ |
| Patient 34 | 44 | Cervical squamous cell carcinoma | Moderately differentiated | IB2 | LN− | LVI+ | PNI− | With AUB | Premenopausal | HPV+ |
| Patient 35 | 51 | Cervical squamous cell carcinoma | Moderately differentiated | IB3 | LN− | LVI+ | PNI− | With AUB | Premenopausal | HPV+ |
| Patient 36 | 52 | Cervical squamous cell carcinoma | Moderately differentiated | IB3 | LN− | LVI+ | PNI+ | With AUB | Premenopausal | HPV+ |
| Patient 37 | 52 | Cervical squamous cell carcinoma | Moderately differentiated | IB3 | LN− | LVI+ | PNI− | With AUB | Premenopausal | HPV+ |
| Patient 38 | 43 | Cervical squamous cell carcinoma | Well-differentiated | IIB | LN− | LVI+ | PNI− | With AUB | Premenopausal | HPV+ |
| Patient 39 | 36 | Cervical squamous cell carcinoma | Poorly differentiated | IIIC1 | LN+ | LVI+ | PNI− | With AUB | Premenopausal | HPV+ |
| Patient 40 | 38 | cervical squamous cell carcinoma | Poorly differentiated | IIIC1 | LN+ | LVI+ | PNI− | With AUB | Premenopausal | HPV+ |
| Patient 41 | 39 | cervical squamous cell carcinoma | Well-differentiated | IIIC1 | LN+ | LVI+ | PNI− | With AUB | Premenopausal | HPV+ |
| Patient 42 | 42 | cervical squamous cell carcinoma | Well-differentiated | IIIC1 | LN+ | LVI+ | PNI− | With AUB | Premenopausal | HPV+ |
| Patient 43 | 29 | cervical squamous cell carcinoma | Moderately differentiated | IIIC1 | LN+ | LVI+ | PNI− | With AUB | Premenopausal | HPV+ |
| Patient 44 | 38 | cervical squamous cell carcinoma | Moderately differentiated | IIIC1 | LN+ | LVI+ | PNI+ | With AUB | Premenopausal | HPV+ |
| Patient 45 | 49 | cervical squamous cell carcinoma | Moderately differentiated | IIIC1 | LN+ | LVI+ | PNI− | With AUB | Premenopausal | HPV+ |
| Patient 46 | 59 | cervical squamous cell carcinoma | Well-differentiated | IA2 | LN− | LVI− | PNI− | Without AUB | Postmenopausal | HPV+ |
| Patient 47 | 58 | cervical squamous cell carcinoma | Moderately differentiated | IA2 | LN− | LVI− | PNI− | Without AUB | Postmenopausal | HPV+ |
| Patient 48 | 58 | cervical squamous cell carcinoma | Poorly differentiated | IB1 | LN− | LVI− | PNI− | Without AUB | Postmenopausal | HPV+ |
| Patient 49 | 54 | cervical squamous cell carcinoma | Moderately differentiated | IB1 | LN− | LVI− | PNI− | Without AUB | Postmenopausal | HPV+ |
| Patient 50 | 59 | cervical squamous cell carcinoma | Moderately differentiated | IB1 | LN− | LVI− | PNI− | Without AUB | Postmenopausal | HPV+ |
| Patient 51 | 49 | cervical squamous cell carcinoma | Poorly differentiated | IB2 | LN− | LVI− | PNI− | Without AUB | Postmenopausal | HPV+ |
| Patient 52 | 62 | cervical squamous cell carcinoma | Moderately differentiated | IB2 | LN− | LVI− | PNI− | Without AUB | Postmenopausal | HPV+ |
| Patient 53 | 63 | cervical squamous cell carcinoma | Well-differentiated | IIA1 | LN− | LVI− | PNI− | Without AUB | Postmenopausal | HPV+ |
| Patient 54 | 67 | cervical squamous cell carcinoma | Well-differentiated | IIA1 | LN− | LVI− | PNI− | Without AUB | Postmenopausal | HPV+ |
| Patient 55 | 56 | cervical squamous cell carcinoma | Poorly differentiated | IB1 | LN− | LVI+ | PNI+ | Without AUB | Postmenopausal | HPV+ |
| Patient 56 | 58 | cervical squamous cell carcinoma | Poorly differentiated | IB2 | LN− | LVI+ | PNI+ | Without AUB | Postmenopausal | HPV+ |
| Patient 57 | 54 | cervical squamous cell carcinoma | Poorly differentiated | IB3 | LN− | LVI+ | PNI− | Without AUB | Postmenopausal | HPV+ |
| Patient 58 | 70 | cervical squamous cell carcinoma | Moderately differentiated | IIIc2 | LN+ | LVI+ | PNI+ | Without AUB | Postmenopausal | HPV+ |
| Patient 59 | 51 | cervical squamous cell carcinoma | Poorly differentiated | IA1 | LN− | LVI− | PNI− | With AUB | Postmenopausal | HPV+ |
| Patient 60 | 57 | cervical squamous cell carcinoma | Well-differentiated | IB1 | LN− | LVI− | PNI− | With AUB | Postmenopausal | HPV+ |
| Patient 61 | 54 | cervical squamous cell carcinoma | Moderately differentiated | IB1 | LN− | LVI− | PNI− | With AUB | Postmenopausal | HPV+ |
| Patient 62 | 74 | cervical squamous cell carcinoma | Moderately differentiated | IB1 | LN− | LVI− | PNI− | With AUB | Postmenopausal | HPV+ |
| Patient 63 | 69 | cervical squamous cell carcinoma | Poorly differentiated | IB2 | LN− | LVI− | PNI+ | With AUB | Postmenopausal | HPV+ |
| Patient 64 | 57 | cervical squamous cell carcinoma | Moderately differentiated | IB2 | LN− | LVI− | PNI− | With AUB | Postmenopausal | HPV+ |
| Patient 65 | 57 | cervical squamous cell carcinoma | Moderately differentiated | IB2 | LN− | LVI− | PNI+ | With AUB | Postmenopausal | HPV+ |
| Patient 66 | 68 | cervical squamous cell carcinoma | Moderately differentiated | IB2 | LN− | LVI− | PNI− | With AUB | Postmenopausal | HPV+ |
| Patient 67 | 57 | cervical squamous cell carcinoma | Well-differentiated | IIA1 | LN− | LVI− | PNI+ | With AUB | Postmenopausal | HPV+ |
| Patient 68 | 64 | cervical squamous cell carcinoma | Well-differentiated | IB2 | LN− | LVI+ | PNI+ | With AUB | Postmenopausal | HPV+ |
| Patient 69 | 68 | cervical squamous cell carcinoma | Moderately differentiated | IB2 | LN− | LVI+ | PNI− | With AUB | Postmenopausal | HPV+ |
| Patient 70 | 62 | cervical squamous cell carcinoma | Moderately differentiated | IB3 | LN− | LVI+ | PNI− | With AUB | Postmenopausal | HPV+ |
| Patient 71 | 68 | cervical squamous cell carcinoma | Well-differentiated | IIA1 | LN− | LVI+ | PNI+ | With AUB | Postmenopausal | HPV+ |
| Patient 72 | 51 | cervical squamous cell carcinoma | Moderately differentiated | IIA1 | LN− | LVI+ | PNI− | With AUB | Postmenopausal | HPV+ |
| Patient 73 | 54 | cervical squamous cell carcinoma | Moderately differentiated | IIA1 | LN− | LVI+ | PNI− | With AUB | Postmenopausal | HPV+ |
| Patient 74 | 62 | cervical squamous cell carcinoma | Moderately differentiated | IIA2 | LN+ | LVI+ | PNI− | With AUB | Postmenopausal | HPV+ |
| Patient 75 | 54 | cervical squamous cell carcinoma | Poorly differentiated | IIIC1 | LN+ | LVI+ | PNI+ | With AUB | Postmenopausal | HPV+ |
| Patient 76 | 68 | cervical squamous cell carcinoma | Poorly differentiated | IIIC1 | LN+ | LVI+ | PNI+ | With AUB | Postmenopausal | HPV+ |
| Patient 77 | 52 | cervical squamous cell carcinoma | Moderately differentiated | IIIC1 | LN+ | LVI+ | PNI+ | With AUB | Postmenopausal | HPV+ |
| Patient 78 | 55 | cervical squamous cell carcinoma | Moderately differentiated | IIIC1 | LN+ | LVI+ | PNI− | With AUB | Postmenopausal | HPV+ |
| Patient 79 | 60 | cervical squamous cell carcinoma | Moderately differentiated | IIIC1 | LN+ | LVI+ | PNI+ | With AUB | Postmenopausal | HPV+ |
| Patient 80 | 61 | cervical squamous cell carcinoma | Moderately differentiated | IIIC1 | LN+ | LVI+ | PNI+ | With AUB | Postmenopausal | HPV+ |
| Patient 81 | 59 | cervical squamous cell carcinoma | Moderately differentiated | IIIc2 | LN+ | LVI+ | PNI+ | With AUB | Postmenopausal | HPV+ |
